# Particle entity in the Doi–Peliti and response field formalisms

**DOI:** 10.1088/1751-8121/acc498

**Published:** 2023-04-11

**Authors:** Marius Bothe, Luca Cocconi, Zigan Zhen, Gunnar Pruessner

**Affiliations:** 1 Department of Mathematics, Imperial College London, SW7 2AZ London, United Kingdom; 2 Department of Genetics and Evolution, University of Geneva, 1205 Geneva, Switzerland; 3 The Francis Crick Institute, NW1 1AT London, United Kingdom

**Keywords:** field theory, particle entity, statistical physics, Doi–Peliti formalism, response field formalism

## Abstract

We introduce a procedure to test a theory for point particle entity, that is, whether said theory takes into account the discrete nature of the constituents of the system. We then identify the mechanism whereby particle entity is enforced in the context of two field-theoretic frameworks designed to incorporate the particle nature of the degrees of freedom, namely the Doi–Peliti field theory and the response field theory that derives from Dean’s equation. While the Doi–Peliti field theory encodes the particle nature at a very fundamental level that is easily revealed, demonstrating the same for Dean’s equation is more involved and results in a number of surprising diagrammatic identities. We derive those and discuss their implications. These results are particularly pertinent in the context of active matter, whose surprising and often counterintuitive phenomenology rests wholly on the particle nature of the agents and their degrees of freedom as particles.

## 1. Introduction

The mathematical description of non-equilibrium many-particle systems typically requires a choice of scale at which their behaviour is resolved. When the focus is on the collective dynamics of a large ensemble of particles, it can be convenient to disregard some of the microscopic information and to rely on a coarse-grained description in terms of densities $\rho(x,t)$, which are continuous in space. What is generally lost upon such coarse-graining is ‘particle entity’, namely the familiar attribute of classical point particles whose initial property of being localised at one point only is preserved under the dynamics, in other words that individual particles can only exist at one position in space at any given time. The distinction between effective and microscopically resolved theories has recently been debated in the context of active matter and, more specifically, entropy production [1–4], where different levels of description grant access to different types of information about the degree of irreversibility of a stochastic process [5]. More generally, the study of sparse collections of interacting particles [6–8] can make it necessary to equip theories with a notion of ‘granularity’ of their constituents. Field theories have traditionally been the most successful approach to capture the physics and mathematics of phenomena emerging from the interaction of many degrees of freedom [9–11]. The Doi–Peliti formalism, which has a discrete number-state master equation as its starting point, is perhaps the best known example of a path-integral approach that preserves particle entity [12, 13]. Another, less familiar example is the response field or Martin–Siggia–Rose–Janssen–De Dominicis [14–16] field theory [17] that derives from Dean’s equation [18–20]. While it is generally accepted that these theories correctly describe the behaviour of physical point particles by construction and that they are, in fact, equivalent [21], the precise mechanism whereby this property is enforced, as well as a general procedure to determine whether a given field theory possesses particle entity, have not been identified. We fill this gap in the following by introducing a signature of particle entity, equation (63), that draws solely on the moments of the integrated number density in a patch Ω of space. These moments can be computed by standard Feynman diagrammatic techniques.

This work is organised as follows. In section 2, we set the scene by introducing the Doi–Peliti field theory and the response field formalism. As an illustrative example, we compute the two-point correlation function of the number density of *n*_0_ non-interacting diffusive particles, thus highlighting some of the key similarities and differences between the two approaches. In section 3 we formalise the concept of single-particle entity and derive different observables to probe it. This signature of particle entity is then applied to the Doi–Peliti field theory (section 4) and the response field formalism of Dean’s equation without interaction (section 5), confirming that both are indeed valid descriptions of physical point particles. In this last section we also discuss the role of integer particle numbers and relate some of the results to a more intuitive probabilistic picture. Finally, in section 6, we summarise our findings and highlight some open questions. Some of the technical details are relegated to the appendices.

## 2. Setting up the formalisms

### 2.1. Doi–Peliti field theory

A Doi–Peliti field theory, sometimes referred to as a coherent-state path integral, is a standard procedure to cast the discrete-state, continuous-time master equation of reaction-diffusion processes in a second quantised form that is amenable to a perturbative treatment [12, 13, 22]. Its derivation starts from the master equation for the probability $P(\{n_i\},t)$ to find the system in state $\{n_i\} = \{n_0,n_1,\ldots\}$, that is to find precisely *n*_*i*_ particles at each site *i*, which is then written in a second quantised form by introducing a Fock space vector $|\{n_i\}\rangle$, together with the ladder operators $a_i^\dagger$ and *a*_*i*_ for creation and annihilation on each lattice site *i*. The operators satisfy the commutation relations

\begin{align*} [a_i,a_j^\dagger] = \delta_{ij}, \quad [a_i,a_j] = [a_i^\dagger,a_j^\dagger] = 0 \end{align*}

and act on $|\{n_i\}\rangle$ according to

\begin{align*} a_j |\{n_i\}\rangle = n_j |\{n_j - 1\} \rangle, \quad a_j^\dagger |\{n_i\}\rangle = |\{n_j + 1\}\rangle ~, \end{align*}

so that $a^\dagger_i a_i$ is the number operator counting the number of particles at site *i*. The notation $\{n_j + 1\}$ and similar is a suggestive shorthand to indicate that this is the same particle number state as $\{n_i\}$ except that the count at site *j* is increased by one. The state of the system is thus described by the mixed state

\begin{align*} | \Psi(t) \rangle = \sum_{\{n_i\}} P(\{n_i\},t) |\{n_j\}\rangle~, \end{align*}

which evolves in time according to an imaginary-time Schrödinger equation of the form [12, 13]

\begin{align*} \partial_t | \Psi(t) \rangle = \hat{A}(a,a^\dagger)| \Psi(t) \rangle~. \end{align*}

For a simple diffusive process on a one-dimensional lattice with homogeneous hopping rate *h* and extinction rate *r*, the operator $\hat{A}$ reads

\begin{align*} \hat{A}(a,a^\dagger) = \sum_i h (a^\dagger_{i+1} + a^\dagger_{i-1} - 2 a^\dagger_{i} )a_i - r (a^\dagger_{i} - 1)a_i ~. \end{align*}

The formal solution of equation (4), $| \Psi(t) \rangle = \mathrm{e}^{\hat{A}t} | \Psi(0) \rangle$, can then be cast into path-integral form, whereby the creation and annihilation operators are converted to time-dependent fields, denoted $\psi_i^\dagger(t)$ and $\psi_i(t)$, respectively. For technical reasons discussed extensively elsewhere [12, 13], it is convenient at this stage to introduce the so-called Doi-shifted creation field, $\tilde{\psi}_i(t)$, according to the convention $\psi_i^\dagger(t) = 1 + \tilde{\psi}_i(t)$. For the case of simple diffusion, equation (5), generalised to *d* dimensions, the action functional of the resulting field theory reads, upon taking the continuum limit,

\begin{align*} A[\tilde{\psi}(\mathbf{x},t),\psi(\mathbf{x},t)] = \int \mathrm{d}^{d}x \mathrm{d}t\, \tilde{\psi}(\mathbf{x},t) ( \partial_t - D \Delta + r) \psi(\mathbf{x},t) \end{align*}

and is fully bilinear. In the continuum limit, the hopping rate *h* and the diffusion constant *D* satisfy $D = \lim_{l \to 0} \, {h}{l^2}$ where *l* is the lattice spacing. In momentum and frequency space it reads

\begin{align*} A[\tilde{\psi}(\mathbf{k},\omega),\psi(\mathbf{k},\omega)] = \int {\mathrm{d}\mkern-8mu^{-}}^d k \mathrm{d}\mkern-8mu^{-} \omega\, \tilde{\psi}(\mathbf{k},\omega) (-\mathring{\imath} \omega + D \mathbf{k}^2 + r) \psi(-\mathbf{k},-\omega) \end{align*}

where we have used the convention

\begin{align*} \psi (\mathbf{x},t) = \int {\mathrm{d}\mkern-8mu^{-}}^d k \mathrm{d}\mkern-8mu^{-} \omega\, \ \mathrm{e}^{\mathring{\imath} \mathbf{k} \cdot \mathbf{x}} \mathrm{e}^{-\mathring{\imath} \omega t} \psi(\mathbf{k},\omega) \quad \textrm{and} \quad \psi (\mathbf{k},\omega) = \int \mathrm{d}^{d}x \mathrm{d}t\, \ \mathrm{e}^{-\mathring{\imath} \mathbf{k} \cdot \mathbf{x}} \mathrm{e}^{\mathring{\imath} \omega t} \psi(\mathbf{x},t) \, , \end{align*}

with ${\mathrm{d}\mkern-8mu^{-}}^d k = \mathrm{d}^{d}k/(2\pi)^d$ and $ \mathrm{d}\mkern-8mu^{-} \omega\, = \mathrm{d}^{d}{\omega}/(2\pi)$ (similarly for $\tilde{\psi})$. We will change freely between different representations.

The diffusive propagator can be obtained by Gaussian integration and reads in $k,\omega$

\begin{align*} \langle \psi(\mathbf{k},\omega) \tilde{\psi}(\mathbf{k}^{^{\prime}},\omega^{^{\prime}}) \rangle = \frac{\delta\mkern-9mu^{-}(\omega+\omega^{^{\prime}}) \delta\mkern-9mu^{-}(\mathbf{k} + \mathbf{k}^{^{\prime}})}{-\mathring{\imath} \omega + D \mathbf{k}^2 + r} \end{align*}

with $\delta\mkern-9mu^{-}(\mathbf{k}) = (2\pi) \delta(\mathbf{k})$ and $\delta\mkern-9mu^{-}(\omega) = (2\pi) \delta(\omega)$. Diagrammatically, the propagator is represented by the Feynman diagram [22]


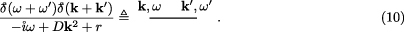


Henceforth we will use the symbol $\overset{\scriptscriptstyle\triangle} = $ to indicate equivalence between diagrams and other mathematical expressions. In all diagrams the arrow of time runs from right to left. Expressing fields in $x,t$, the propagator reads

\begin{align*} \langle \psi(\mathbf{x},t) \tilde{\psi}(\mathbf{x}^{^{\prime}},t^{^{\prime}})\rangle = \theta(t-t^{^{\prime}}) \left(\frac{1}{4\pi D (t-t^{^{\prime}})}\right)^{d/2}\operatorname{exp}\left(-\frac{(\mathbf{x}-\mathbf{x}^{^{\prime}})^2}{4D(t-t^{^{\prime}})}\right) ~, \end{align*}

for $r \to 0^+$, with the Heaviside theta function $\theta(t)$ enforcing causality. The extinction rate *r* has solely the role to establish causality, as it generates the Heaviside theta function, and regularize the large *t* behaviour, as it guarantees that any density vanishes in the large *t* limit. In the following, we may take the limit $r \to 0^+$ whenever convenient. For completeness, the propagator in mixed momentum-time representation reads

\begin{align*} \langle \psi(\mathbf{k},t) \tilde{\psi}(\mathbf{k}^{^{\prime}},t^{^{\prime}}) \rangle = \theta(t-t^{^{\prime}}) \delta\mkern-9mu^{-}(\mathbf{k}+\mathbf{k}^{^{\prime}}) \mathrm{e}^{-D k^2 (t-t^{^{\prime}})} ~. \end{align*}

A general observable in the Fock space system is given as a function of occupation numbers $\mathcal{O}(\{n_i\})$. In the Doi–Peliti formalism this corresponds to an operator $\hat{\mathcal{O}}^{^{\prime}}(\{a_i^\dagger a_i\})$, which is defined by acting on the pure state $ | \{n_i\} \rangle$ according to $\hat{\mathcal{O}}^{^{\prime}}(\{a_i^\dagger a_i\}) | \{n_i\} \rangle = \mathcal{O}(\{n_i\}) | \{n_i\} \rangle$. We can use the commutation relation (1) to normal-order $\hat{\mathcal{O}}^{^{\prime}}(\{a_i^\dagger a_i\})$ such that all annihilation operators are on the right and all creation operators are on the left. We define the normal ordered operator that acts identical to $\hat{\mathcal{O}}^{^{\prime}}(\{a_i^\dagger a_i\})$ as $\hat{\mathcal{O}}(\{a_i^\dagger\},\{a_i\})$. Its expectation translates into a path integral according to the following procedure [23]

\begin{align*} \langle \mathcal{O} \rangle &amp;= \sum_{\{n_i\}} \mathcal{O}(\{n_i\}) P(\{n_i\},t) |\{n_j\}\rangle \end{align*}

\begin{align*} &amp; \quad\;\; = \langle \unicode{x263C} | \hat{\mathcal{O}}(\{a_i^\dagger\},\{a_i\}) \mathrm{e}^{\tilde{A}t}| \Psi(0) \rangle \end{align*}

\begin{align*} &amp; \quad\;\; = \int \mathcal{D} \psi \mathcal{D} \tilde{\psi} \ \hat{\mathcal{O}}(\{\tilde{\psi}_i(t) + 1\},\{\psi_i(t)\}) \ \mathrm{e}^{A[\tilde{\psi},\psi] } \mathbb{I}(\tilde{\psi}(0) + 1) \end{align*}

where we have introduced the coherent state,

\begin{align*} \langle \unicode{x263C} | = \sum_{\{n_i\}} \langle \{n_i\} | \end{align*}

with $\sum_{\{n_i\}} \langle \{n_i\}|$ summing over all *n*-particle occupation number states, as well as the initialisation operator $\mathbb{I}(a_i^\dagger)$, which satisfies $\mathbb{I}(a_i^\dagger)|0\rangle = | \Psi(0) \rangle$, with $| 0 \rangle$ the vacuum state. Going to equation (15) the normal ordering of the operator $\hat{\mathcal{O}}(\{a_i\},\{a_i^\dagger\})$ allowed us to replace annihilation and creation operators by the right and left eigenvalues of the coherent states $\Psi_i$ and $\tilde{\Psi}_i+1$ respectively.

For an initial condition where *m*_*i*_ particles are placed at each site *i* at time *t* = 0, the initialisation appears within the path integral equation (15) as

\begin{align*} \mathbb{I}(\tilde{\psi}(0) + 1) = \prod_i (\tilde{\psi}_i(0) + 1)^{m_i} = \prod_i \sum_{k=0}^{m_i} {m_i \choose k} \tilde{\psi}_i^k(0) \end{align*}

after the Doi shift. The process can also be initialised in a statistical mixture of occupation number states, e.g. where the occupation number at each site is drawn from a Poisson distribution with mean *q*,

\begin{align*} \mathbb{I}_\mathrm{Poiss}(\tilde{\psi}(0) + 1) = \prod_i \left[ \sum_{k=0}^\infty \left( \frac{\mathrm{e}^{- q} q^k }{k!}\right)(\tilde{\psi}_i(0) + 1)^{k} \right] = \prod_i \mathrm{e}^{q \tilde{\psi}_i(0)}~. \end{align*}

### 2.2. Dean’s equation in the response field formalism

Dean’s equation [18] is a stochastic differential equation of the Itô type obeyed by the number density function $\rho(\mathbf{x},t)$ for a system of Langevin processes interacting via a pairwise potential. It is an exact mapping of, and thus contains the same information as, the full set of Langevin equations for the individual ‘single particle’ processes. It reads

\begin{align*} \partial_t \rho (\mathbf{x},t) = \nabla \cdot \left( \rho \nabla \left. \frac{\delta F[\rho]}{\delta \rho}\right|_{\rho(\mathbf{x},t)} \right) + \nabla \cdot(\rho^{1/2}\boldsymbol{\eta}(\mathbf{x},t)) + \sum_i n_i \delta(t-t_i) \delta(\mathbf{x}-\mathbf{x}_i) \end{align*}

where $F[\rho]$ denotes the free energy functional, defined as

\begin{align*} F[\rho(\mathbf{x})] = \int \mathrm{d}^{d}x \rho(\mathbf{x}) \left( V(\mathbf{x}) + D \log(\rho(\mathbf{x})) + \frac{1}{2}\int \mathrm{d}^{d}y U(\mathbf{x}-\mathbf{y}) \rho(y) \right) ~, \end{align*}

with $V(\mathbf{x})$ a general single-particle potential and $U(\mathbf{x}-\mathbf y)$ a translationally invariant pairwise interaction potential. The term $D \log(\rho)$in equation (20) is the additional second derivative from Ito’s lemma rewritten as an entropic contribution to the free energy. The last term on the right-hand side of equation (19) describes the initialisation of $n_i \in \mathbb{Z}$ particles in state **x**_*i*_ at time *t*_*i*_ so that $\lim_{t\to-\infty} \rho(\mathbf{x},t) = 0$. The vector-valued noise $\boldsymbol{\eta}(\mathbf{x},t) \in \mathbb{R}^d$ is an uncorrelated white noise with covariance

\begin{align*} \langle \eta_\mu(\mathbf{x},t) \eta_\nu(\mathbf{x}^{^{\prime}},t^{^{\prime}})\rangle = 2 D \delta_{\mu \nu}\delta(t-t^{^{\prime}})\delta(\mathbf{x}-\mathbf{x}^{^{\prime}}) ~, \end{align*}

for $\mu,\nu = 1,2,\ldots,d$. The unique feature of Dean’s formalism is the nature of the noise term in equation (19), $\nabla \cdot(\rho^{1/2} \boldsymbol{\eta})$, which is both conservative and Itô-multiplicative, thus conserving the total particle number while preventing fluctuations from producing regions of negative density. Following the standard procedure [11, 17], which requires special attention due to the multiplicative nature of the noise [20, 24], Dean’s equation (19) for the time and space dependent field $\rho(\mathbf{x},t)$ can be cast as a response field, or Martin–Siggia–Rose–Janssen–De Dominicis, field theory with action

\begin{align*} A[\rho,\tilde{\rho}]=\int \mathrm{d}^{d}x \mathrm{d}t\, \tilde\rho\left( \partial_t \rho-\nabla\cdot\rho\nabla \left. \frac{\delta F[\rho]}{\delta \rho}\right|_{\rho(\mathbf{x},t)} \right)-\rho D(\nabla \tilde \rho)^2 - \tilde{\rho} \sum_i n_i \delta(t-t_i) \delta(\mathbf{x}-\mathbf{x}_i), \nonumber\\ \end{align*}

which simplifies to

\begin{align*} A[\rho,\tilde{\rho}] &amp;=\int \mathrm{d}^{d}x \mathrm{d}t\, \tilde\rho(\mathbf{x},t)\left( \partial_t \rho(\mathbf{x},t) -D \Delta \rho(\mathbf{x},t) \right) - \tilde{\rho} \sum_i n_i \delta(t-t_i) \delta(\mathbf{x}-\mathbf{x}_i) -\rho D(\nabla \tilde \rho)^2 \end{align*}

\begin{align*} &amp; \qquad\;\;\; = \int {\mathrm{d}\mkern-8mu^{-}}^d k \mathrm{d}\mkern-8mu^{-} \omega\, \tilde{\rho}(-\mathbf{k},-\omega) (-\mathring{\imath} \omega + D \mathbf{k}^2) \rho(\mathbf{k},\omega) - \tilde{\rho}(\mathbf{k},\omega) \sum_i n_i \mathrm{e}^{\mathring{\imath} \mathbf{k} \cdot \mathbf{x}_i} \mathrm{e}^{-\mathring{\imath} \omega t_i} \nonumber \\ &amp;\qquad\quad\;\;\; + \int {\mathrm{d}\mkern-8mu^{-}}^d k {\mathrm{d}\mkern-8mu^{-}}^d k^{^{\prime}} \mathrm{d}\mkern-8mu^{-} \omega\, \mathrm{d}\mkern-8mu^{-} \omega^{^{\prime}}\, D (\mathbf{k} \cdot \mathbf{k}^{^{\prime}}) \tilde{\rho}(\mathbf{k},\omega) \tilde{\rho}(\mathbf{k}^{^{\prime}},\omega^{^{\prime}}) \rho(-(\mathbf{k}+\mathbf{k}^{^{\prime}}),-(\omega+\omega^{^{\prime}})) \end{align*}

in the case of non-interacting particles undergoing simple diffusion without external potential. Unlike the Doi–Peliti path integral, equation (15), the initialisation here shows up as a term in the action. In a diagrammatic perturbation theory, these *n*_*i*_ particles starting from positions **x**_*i*_ will be shown as a small, filled circle acting as a source:


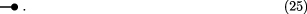


We will make the simplifying assumption of having only a single non-zero *n*_*i*_, namely *n*_0_, and generalise our result in appendix B. The presence of the source spoils translational invariance and as a result, the hallmark *δ*-function as it normally multiplies any correlation function, say $\delta\mkern-9mu^{-}(\mathbf{k}_0+\mathbf{k}_1+\ldots+\mathbf{k}_n)$ will be replaced by

\begin{align*} \int{\mathrm{d}\mkern-8mu^{-}}^d k_0 \operatorname{e}^{\mathring{\imath}\mathbf{k}_0\cdot\mathbf{x}_0} \delta(\mathbf{k}_0+\mathbf{k}_1+\ldots+\mathbf{k}_n) = \operatorname{e}^{\mathring{\imath}\mathbf{k}_1+\ldots+\mathbf{k}_n\cdot\mathbf{x}_0} \ \end{align*}

where readability is improved by it, we will retain the integral.

The expectation value of a field-dependent observable $\mathcal{O}[\rho]$ can then be computed via the path integral

\begin{align*} \langle \mathcal{O}[\rho] \rangle = \int \mathcal{D}\rho \mathcal{D} \tilde{\rho} \ \mathcal{O}[\rho] \operatorname{e}^{- A[\rho,\tilde{\rho}]} \end{align*}

where $\tilde{\rho}$ is the purely imaginary response field. The normalisation is chosen such that $\langle 1 \rangle = 1$. The action *A* is then split into a bilinear and an interacting part, denoted *A*_0_ and $A_\mathrm{int}$ respectively, according to

\begin{align*} A_0[\rho,\tilde{\rho}] = \int \mathrm{d}^{d}x \mathrm{d}t\, \tilde\rho\left( \partial_t \rho-D \Delta \rho \right) \end{align*}

and

\begin{align*} A_\mathrm{int}[\rho,\tilde{\rho}]&amp; = - \int \mathrm{d}^{d}x \mathrm{d}t\, \Bigg\{\rho(\mathbf{x},t) D(\nabla \tilde \rho(\mathbf{x},t))^2 \nonumber \\ &amp; \quad + \tilde{\rho}(\mathbf{x},t) \nabla_{\mathbf{x}} \cdot \left( \rho(\mathbf{x},t) \nabla_{\mathbf{x}} \left[ V(\mathbf{x}) + \int \mathrm{d}^{d}y \ \frac{1}{2}U(\mathbf{x}-\mathbf{y}) \rho(\mathbf{y},t) \right] \right)\nonumber\\ &amp; \quad + \tilde{\rho}(\mathbf{x},t) \sum_i n_i \delta(\mathbf{x}-\mathbf{x}_i) \delta(t-t_i) \Bigg\} \end{align*}

\begin{align*} &amp;= \int {\mathrm{d}\mkern-8mu^{-}}^d k_{1,2,3}\,\, \mathrm{d}\mkern-8mu^{-} \omega_{1,2,3}\,\delta\mkern-9mu^{-}(\mathbf{k}_1+\mathbf{k}_2+\mathbf{k}_3)\delta\mkern-9mu^{-}(\omega_1+\omega_2+\omega_3)\nonumber \\ &amp;\quad \times \bigg\{ \rho(\mathbf{k}_1,\omega_1) D (\mathbf{k}_2\cdot\mathbf{k}_3)\tilde{\rho}(\mathbf{k}_2,\omega_2)\tilde{\rho}(\mathbf{k}_3,\omega_3)\nonumber \\ &amp;\quad + \tilde{\rho}(\mathbf{k}_1,\omega_1) ((\mathbf{k}_2+\mathbf{k}_3)\cdot\mathbf{k}_3)\rho(\mathbf{k}_2,\omega_2)\bigg[V(\mathbf{k}_3)\delta\mkern-9mu^{-}(\omega_3) + \frac{1}{2}U(\mathbf{k}_3)\rho(\mathbf{k}_3,\omega_3) \bigg] \bigg\}\nonumber \\ &amp; \quad- \int {\mathrm{d}\mkern-8mu^{-}}^d k\, \mathrm{d}\mkern-8mu^{-} \omega\, \tilde{\rho}(\mathbf{k},\omega) \sum_i n_i \operatorname{e}^{\mathring{\imath}\mathbf{k}\cdot\mathbf{x}_i} \operatorname{e}^{-\mathring{\imath}\omega t_i}. \end{align*}

Finally, expectations are computed in a perturbation theory about the bilinear theory using

\begin{align*} \langle \mathcal{O}[\rho] \rangle = \sum_{n=0}^\infty \left\langle \frac{\left(- A_\mathrm{int}[\rho,\tilde{\rho}] \right)^n}{n!} \mathcal{O}[\rho] \right\rangle_0 ~, \end{align*}

where

\begin{align*} \langle\bullet\rangle_0=\int\mathcal{D} \rho\,\mathcal{D} \tilde{\rho}\, \bullet \operatorname{e}^{-A_0[\rho,\tilde{\rho}]} \end{align*}

denotes expectation with respect to the bilinear action, equation (28). The right hand side of equation (31) involves products of fields and the Wick–Isserlis theorem [9] can be invoked to express these in terms of the bare propagator,


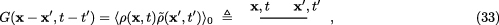


obtained from the bilinear action. In *d* dimensions, the bare propagator reads

**Figure 1.**
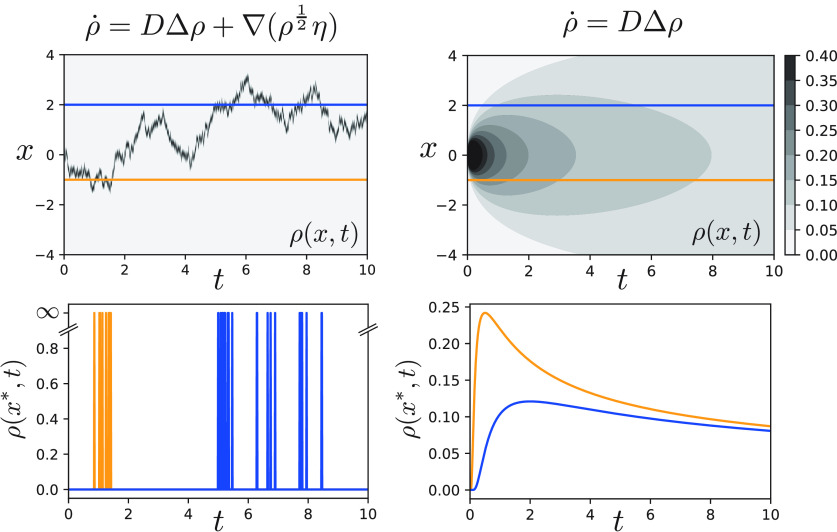
The time-dependent number density $\rho(x,t)$ for a physical point particle undergoing diffusion is expected to remain localised under the dynamics, indicating that the particle can only occupy one position in space at any given time. While this property is preserved under Dean’s dynamics (left column), it is generally lost when resorting to effective descriptions, such as the classical diffusion equation (right column). This difference is most obvious when measuring the instantaneous particle number density at two points a finite distance away from each other (bottom row).

\begin{align*} G(\mathbf{x}-\mathbf{x}^{^{\prime}},t-t^{^{\prime}}) = \theta(t-t^{^{\prime}}) \left( \frac{1}{4\pi D (t-t^{^{\prime}})}\right)^{d/2} \operatorname{exp}\left(-\frac{(\mathbf{x}-\mathbf{x}^{^{\prime}})^2}{4D(t-t^{^{\prime}})}\right), \end{align*}

with the Heaviside theta function $\theta(t)$ enforcing causality. This propagator is identical to that of the corresponding Doi–Peliti field theory, equation (11). For later use, we recall the form of the propagator in momentum-frequency representation,

\begin{align*} \langle \rho(\mathbf{k},\omega) \tilde{\rho}(\mathbf{k}^{^{\prime}},\omega^{^{\prime}}) \rangle_0 = \frac{\delta(\mathbf{k}+\mathbf{k}^{^{\prime}}) \delta(\omega+\omega^{^{\prime}})}{-\mathring{\imath} \omega + D \mathbf{k}^2+r} ~, \end{align*}

which we have amended by the extinction rate $r \to 0^+$ to enforce causality, as equation (9). Further, we introduce the mixed momentum-time representation,

\begin{align*} \langle \rho(\mathbf{k},t) \tilde{\rho}(\mathbf{k}^{^{\prime}},t^{^{\prime}})\rangle_0 = \theta(t-t^{^{\prime}}) \delta(\mathbf{k}+\mathbf{k}^{^{\prime}}) \operatorname{e}^{-D (t-t^{^{\prime}})\mathbf{k}^2} ~, \end{align*}

see equation (12). For non-interacting particles in a flat potential, $\nabla V(\mathbf{x}) = 0$, $\nabla U(\mathbf{x}) = 0$, the bare propagator equals the full propagator,

\begin{align*} \langle \rho(\mathbf{k},t) \tilde{\rho}(\mathbf{k}^{^{\prime}},t^{^{\prime}})\rangle = \langle \rho(\mathbf{k},t) \tilde{\rho}(\mathbf{k}^{^{\prime}},t^{^{\prime}})\rangle_0 \end{align*}

as the only non-linear term in the action is the amputated three-point vertex


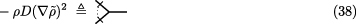


with the dashes on the propagators denoting spatial derivatives acting on the response fields and the dotted line the scalar product of these derivatives. The presence of such a vertex in the free particle case is a non-trivial feature of Dean’s equation and clashes somewhat with the notion of ‘interaction’ associated with terms of order higher than bilinear [11]. As we will demonstrate below, equation (38), which we will refer to interchangeably as *Dean’s vertex* or a *virtual branching* vertex, is the term that implements the particle nature of the degrees of freedom within the Dean framework. In contrast to Doi–Peliti, particle entity in the response field formalism of Dean’s equation is a perturbative feature. The effect of Dean’s vertex is illustrated in figure 1 by comparison with the standard diffusion equation, which lacks particle entity.

The Doi–Peliti field theory and the response field theory derived from Dean’s equation can be mapped onto each other by means of a Cole–Hopf transformation of the fields [21],

\begin{align*} \psi^\dagger \to \mathrm{e}^{\tilde\rho}, \quad \psi \to \rho \mathrm{e}^{-\tilde{\rho}}~. \end{align*}

This equivalence implies that the two formalisms should be equally capable of capturing particle entity. The precise mechanisms by which each does so, however, turn out to be very different, as we will see in detail in sections 4 and 5.

### 2.3. Example: the two-point density correlation function

To illustrate the similarities and differences between the two formalisms introduced above, we now calculate the two-point correlation function of the particle number density for *n*_0_ non-interacting diffusive particles in a flat potential, $\nabla V(\mathbf{x}) = 0$ and $\nabla U(\mathbf{x}) = 0$, all initialised at the same position *x*_0_ and time *t*_0_, first in the Doi–Peliti scheme and then using Dean’s equation. While the result of this detailed calculation is somewhat trivial and can be derived by straightforward probabilistic arguments, its derivation elucidates certain formalism-specific cancellation mechanisms that will play an important role in the remainder of this work. The reader interested in the generic definition of particle entity but not in the details of the field theoretic approach can skip directly to section 3.

We first use the parameterisation of the field theories in **k** and *ω*, which is very commonly used in field theories. In real-space and time, the two-point correlation function $C(\mathbf{x}_1,\mathbf{x}_2,t_1,t_2)$ in the Doi–Peliti framework is the observable [11, 12]

\begin{align*} C(\mathbf{x}_1,\mathbf{x}_2, t_1, t_2) &amp; = {\left\langle \big(\psi^{\dagger}(\mathbf{x}_2,t_2) \psi(\mathbf{x}_2,t_2)\big) \big(\psi^{\dagger}(\mathbf{x}_1,t_1) \psi(\mathbf{x}_1,t_1)\big) \psi^{\dagger n_0}(\mathbf{x}_0,t_0) \right\rangle} \end{align*}

\begin{align*} &amp; \qquad = {n_0 \choose 1} {\left\langle \psi(\mathbf{x}_2,t_2)\tilde{\psi}(\mathbf{x}_1,t_1) \right\rangle} {\left\langle \psi(\mathbf{x}_1,t_1)\tilde{\psi}(\mathbf{x}_0,t_0) \right\rangle} \end{align*}

\begin{align*} &amp;\qquad \quad + {n_0 \choose 1} {\left\langle \psi(\mathbf{x}_1,t_1)\tilde{\psi}(\mathbf{x}_2,t_2) \right\rangle}{\left\langle \psi(\mathbf{x}_2,t_2)\tilde{\psi}(\mathbf{x}_0,t_0) \right\rangle}\\ &amp;\qquad \quad + 2 {n_0 \choose 2} {\left\langle \psi(\mathbf{x}_2,t_2)\tilde{\psi}(\mathbf{x}_0,t_0) \right\rangle}{\left\langle \psi(\mathbf{x}_1,t_1)\tilde{\psi}(\mathbf{x}_0,t_0) \right\rangle} \end{align*}

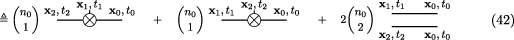


where we assume $\mathbf{x}_1\ne\mathbf{x}_2$ to avoid the special case of non-commutation of the operators. The high number of terms in equation (40) is due to the Doi-shift, which splits each daggered creator field in two terms, $\psi^{\dagger} = 1+\tilde{\psi}$. This turns the contribution of the initial particles into $\psi^{\dagger n_0} = \sum_k^{n_0} {n_0 \choose k} \tilde{\psi}^{n_0}$. The vertices made from a crossed circle in equation (42) are meant to indicate an annihilation field at the indicated position and time with immediate re-creation. Equation (40) has the generic form of a two-point correlation function in the Doi–Peliti framework without interaction.

Equation (41) is still expressed in real space and direct time and needs to be Fourier-transformed to write it in the common $\mathbf{k},\omega$ parameterisation. Each of the three terms in equation (41) requires four integrals in **k** and four in *ω*, for example


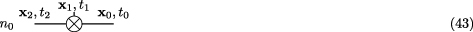

\begin{align*} &amp;\overset{\scriptscriptstyle\triangle} = n_0 \int {\mathrm{d}\mkern-8mu^{-}}^d k_2{\mathrm{d}\mkern-8mu^{-}}^d k^{^{\prime}}_1{\mathrm{d}\mkern-8mu^{-}}^d k_1{\mathrm{d}\mkern-8mu^{-}}^d k_0 \mathrm{d}\mkern-8mu^{-} \omega_2\, \mathrm{d}\mkern-8mu^{-} \omega^{^{\prime}}_1\, \mathrm{d}\mkern-8mu^{-} \omega_1\, \mathrm{d}\mkern-8mu^{-} \omega_0\, \frac{\delta\mkern-9mu^{-}(\mathbf{k}_2+\mathbf{k}^{^{\prime}}_1)\delta\mkern-9mu^{-}(\omega_2+\omega^{^{\prime}}_1)}{-\mathring{\imath}\omega_2+D\mathbf{k}_2^2+r} \frac{\delta\mkern-9mu^{-}(\mathbf{k}_1+\mathbf{k}_0) \delta\mkern-9mu^{-}(\omega_1+\omega_0)} {-\mathring{\imath}\omega_1+D\mathbf{k}_1^2+r}\nonumber\\ &amp; \quad \times\operatorname{e}^{\mathring{\imath} (\mathbf{k}_2\cdot\mathbf{x}_2 + \mathbf{k}^{^{\prime}}_1\cdot\mathbf{x}_1 + \mathbf{k}_1\cdot\mathbf{x}_1 + \mathbf{k}_0\cdot\mathbf{x}_0) } \operatorname{e}^{-\mathring{\imath} (\omega_2 t_2 + \omega^{^{\prime}}_1 t_1 + \omega_1 t_1 + \omega_0 t_0) } \end{align*}

drawing on the propagator introduced in equation (9). Using the *δ*-functions, the integrals in each term are immediately reduced to only two, all differing solely in the arguments of the exponentials:

\begin{align*} C(\mathbf{x}_1,\mathbf{x}_2, t_1, t_2)&amp; = \int {\mathrm{d}\mkern-8mu^{-}}^d k_2{\mathrm{d}\mkern-8mu^{-}}^d k_1 \mathrm{d}\mkern-8mu^{-} \omega_2\, \mathrm{d}\mkern-8mu^{-} \omega_1\, \frac{1}{-\mathring{\imath}\omega_2+D\mathbf{k}_2^2+r} \frac{1} {-\mathring{\imath}\omega_1+D\mathbf{k}_1^2+r}\nonumber\\ &amp;\quad \times \Big\{n_0 \operatorname{e}^{\mathring{\imath} (\mathbf{k}_2\cdot(\mathbf{x}_2-\mathbf{x}_1) + \mathbf{k}_1\cdot(\mathbf{x}_1-\mathbf{x}_0) ) } \operatorname{e}^{-\mathring{\imath} (\omega_2 (t_2-t_1) + \omega_1 (t_1-t_0) ) }\nonumber\\ &amp;\quad + n_0 \operatorname{e}^{\mathring{\imath} (\mathbf{k}_2\cdot(\mathbf{x}_2-\mathbf{x}_0) + \mathbf{k}_1\cdot(\mathbf{x}_1-\mathbf{x}_2) ) } \operatorname{e}^{-\mathring{\imath} (\omega_2 (t_2-t_0) + \omega_1 (t_1-t_2) ) }\nonumber\\ &amp; \quad + n_0(n_0-1) \operatorname{e}^{\mathring{\imath} (\mathbf{k}_2\cdot(\mathbf{x}_2-\mathbf{x}_0) + \mathbf{k}_1\cdot(\mathbf{x}_1-\mathbf{x}_0) ) } \operatorname{e}^{-\mathring{\imath} (\omega_2 (t_2-t_0) + \omega_1 (t_1-t_0) ) } \Big\} \end{align*}

with $r\to 0^+$ still to be taken. The first of the three terms in the integrand describes the propagation of any of *n*_0_ particles from **x**_0_ at *t*_0_ to **x**_1_ at *t*_1_ and from there to **x**_2_ at *t*_2_. This term will contribute only if $t_2\geqslant t_1\geqslant t_0$. The second term describes a similar process, from **x**_0_ at *t*_0_ to **x**_2_ at *t*_2_ and from there to **x**_1_ at *t*_1_, contributing only if $t_1\geqslant t_2\geqslant t_0$. The last term describes the propagation of two independent particles from **x**_0_ at *t*_0_ to **x**_1_ at *t*_1_ and another one from **x**_0_ at *t*_0_ to **x**_2_ at *t*_2_. There are $n_0(n_0-1)$ such pairs. If $n_0\leqslant 1$, the last term vanishes, leaving only the first two terms, both of which vanish if $t_1 = t_2$ and $\mathbf{x}_1\ne \mathbf{x}_2$ as we will show below, because a *particle* cannot possibly be found at two different places simultaneously. Equation (45) completes the derivation of the correlation function in the Doi–Peliti framework.

To derive the correlation function in Dean’s framework, we use the action as stated in equation (23) with both the interaction and the source treated perturbatively. The role of the creator fields in the field theory of Dean’s equation is very different from Doi–Peliti. In the Dean framework, the two-point correlation function is


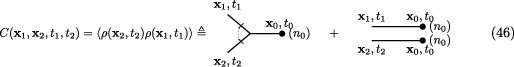


as every field $\rho(\mathbf{x},t)$ can be matched with a creator field from the perturbative part of the action, shown as a small filled circle at the right end of the incoming propagators. Each such creator field appears with a *coupling* *n*_0_, which we have highlighted by writing it in brackets behind each source in the diagram. While the second term in equation (46) is structurally identical to the last term in equation (42) and indeed captures the same process, the pre-factors of the two differ by *n*_0_. The first two terms in equation (42) on the other hand seem to be absent from equation (46). In turn, the first diagram of equation (46), is solely due to the Dean-vertex equation (38) and therefore absent in Doi–Peliti, equation (42). Writing this term in $\mathbf{k}, \omega$ gives


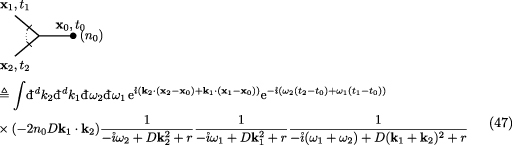


using equation (35) for the propagator and where the factor $(-2 n_0 D \mathbf{k}_1\cdot\mathbf{k}_2)$ is due to the sign of the interaction term in the action equation (23), including a factor 2 from symmetry.

The second term in equation (46) can be read off from equations (42) and (45). Its pre-factor of $n_0^2$ has to be split into $n_0^2 = n_0(n_0-1)+n_0$ to reveal the cancellation mechanism,


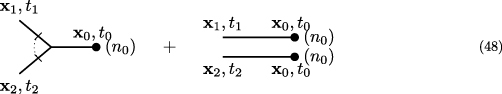

\begin{align*} \overset{\scriptscriptstyle\triangle} = &amp;\int {\mathrm{d}\mkern-8mu^{-}}^d k_2{\mathrm{d}\mkern-8mu^{-}}^d k_1 \mathrm{d}\mkern-8mu^{-} \omega_2\, \mathrm{d}\mkern-8mu^{-} \omega_1\, \operatorname{e}^{\mathring{\imath} (\mathbf{k}_2\cdot (\mathbf{x}_2-\mathbf{x}_0) + \mathbf{k}_1\cdot (\mathbf{x}_1-\mathbf{x}_0) ) } \operatorname{e}^{-\mathring{\imath} (\omega_2 (t_2 - t_0) + \omega_1 (t_1 - t_0) ) }\times \frac{1}{-\mathring{\imath}\omega_2+D\mathbf{k}_2^2+r} \nonumber\\ &amp; \quad \times \frac{1}{-\mathring{\imath}\omega_1+D\mathbf{k}_1^2+r} \left( \frac{-2 n_0 D \mathbf{k}_1\cdot\mathbf{k}_2}{-\mathring{\imath}(\omega_1+\omega_2)+D(\mathbf{k}_1+\mathbf{k}_2)^2+r} +n_0 + n_0(n_0-1) \right) \end{align*}

\begin{align*} &amp; \quad = \int {\mathrm{d}\mkern-8mu^{-}}^d k_2{\mathrm{d}\mkern-8mu^{-}}^d k_1 \mathrm{d}\mkern-8mu^{-} \omega_2\, \mathrm{d}\mkern-8mu^{-} \omega_1\, \operatorname{e}^{\mathring{\imath} (\mathbf{k}_2\cdot (\mathbf{x}_2-\mathbf{x}_0) + \mathbf{k}_1\cdot (\mathbf{x}_1-\mathbf{x}_0) ) } \operatorname{e}^{-\mathring{\imath} (\omega_2 (t_2 - t_0) + \omega_1 (t_1 - t_0) ) }\nonumber\\ &amp; \quad \qquad \times \Bigg\{n_0\frac{1}{-\mathring{\imath}(\omega_1+\omega_2)+D(\mathbf{k}_1+\mathbf{k}_2)^2+r}\nonumber\\ &amp; \quad \qquad\times \left( \frac{1}{-\mathring{\imath}\omega_2+D\mathbf{k}_2^2+r}+ \frac{1}{-\mathring{\imath}\omega_1+D\mathbf{k}_1^2+r}- \frac{r }{(-\mathring{\imath}\omega_1+D\mathbf{k}_1^2+r)(-\mathring{\imath}\omega_2+D\mathbf{k}_2^2+r)} \right)\nonumber\\ &amp; \quad \qquad + n_0(n_0-1) \frac{1}{-\mathring{\imath}\omega_2+D\mathbf{k}_2^2+r} \frac{1}{-\mathring{\imath}\omega_1+D\mathbf{k}_1^2+r}\Bigg\}. \end{align*}

The term proportional to *r* in the numerator eventually vanishes when *r* → 0. To see now that equation (50) is in fact identical to the first two terms in equations (42) and (45) requires a simple substitution of the dummy variables, for example $\mathbf{k}_1+\mathbf{k}_2$ becoming **k**_1_,

\begin{align*} &amp;\int {\mathrm{d}\mkern-8mu^{-}}^d k_2{\mathrm{d}\mkern-8mu^{-}}^d k_1 \mathrm{d}\mkern-8mu^{-} \omega_2\, \mathrm{d}\mkern-8mu^{-} \omega_1\, \operatorname{e}^{\mathring{\imath} (\mathbf{k}_2\cdot (\mathbf{x}_2-\mathbf{x}_0) + \mathbf{k}_1\cdot (\mathbf{x}_1-\mathbf{x}_0) ) } \operatorname{e}^{-\mathring{\imath} (\omega_2 (t_2 - t_0) + \omega_1 (t_1 - t_0) ) }\nonumber\\ &amp;\qquad\times \frac{1}{-\mathring{\imath}(\omega_1+\omega_2)+D(\mathbf{k}_1+\mathbf{k}_2)^2+r} \frac{1}{-\mathring{\imath}\omega_2+D\mathbf{k}_2^2+r} \end{align*}

\begin{align*} &amp; \quad = \int {\mathrm{d}\mkern-8mu^{-}}^d k_2{\mathrm{d}\mkern-8mu^{-}}^d k_1 \mathrm{d}\mkern-8mu^{-} \omega_2\, \mathrm{d}\mkern-8mu^{-} \omega_1\, \operatorname{e}^{\mathring{\imath} (\mathbf{k}_2\cdot (\mathbf{x}_2-\mathbf{x}_1) + \mathbf{k}_1\cdot (\mathbf{x}_1-\mathbf{x}_0) ) } \operatorname{e}^{-\mathring{\imath} (\omega_2 (t_2 - t_1) + \omega_1 (t_1 - t_0) ) }\nonumber\\ &amp;\qquad\times \frac{1}{-\mathring{\imath}\omega_1+D\mathbf{k}_1^2+r} \frac{1}{-\mathring{\imath}\omega_2+D\mathbf{k}_2^2+r} ~. \end{align*}

In summary, after pairing in equation (50) the interaction term of Dean’s equation with the two independent propagators, the field theory of Dean’s equation reproduces the two-point correlation function as the Doi–Peliti framework, equation (45), except for a term proportional to *r* which vanishes in the limit of *r* → 0:


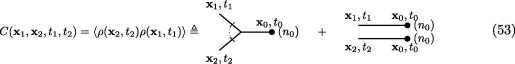

\begin{align*} \overset{\scriptscriptstyle\triangle} = &amp;\;\int {\mathrm{d}\mkern-8mu^{-}}^d k_2{\mathrm{d}\mkern-8mu^{-}}^d k_1 \mathrm{d}\mkern-8mu^{-} \omega_2\, \mathrm{d}\mkern-8mu^{-} \omega_1\, \frac{1}{-\mathring{\imath}\omega_2+D\mathbf{k}_2^2+r} \frac{1} {-\mathring{\imath}\omega_1+D\mathbf{k}_1^2+r}\nonumber\\ &amp; \times \Big\{n_0 \operatorname{e}^{\mathring{\imath} (\mathbf{k}_2\cdot(\mathbf{x}_2-\mathbf{x}_1) + \mathbf{k}_1\cdot(\mathbf{x}_1-\mathbf{x}_0) ) } \operatorname{e}^{-\mathring{\imath} (\omega_2 (t_2-t_1) + \omega_1 (t_1-t_0) ) }\nonumber\\ &amp; + n_0 \operatorname{e}^{\mathring{\imath} (\mathbf{k}_2\cdot(\mathbf{x}_2-\mathbf{x}_0) + \mathbf{k}_1\cdot(\mathbf{x}_1-\mathbf{x}_2) ) } \operatorname{e}^{-\mathring{\imath} (\omega_2 (t_2-t_0) + \omega_1 (t_1-t_2) ) } \nonumber\\ &amp; - n_0 \frac{r}{-\mathring{\imath}(\omega_1+\omega_2)+D(\mathbf{k}_1+\mathbf{k}_2)^2+r}\nonumber\\ &amp; \times \operatorname{e}^{\mathring{\imath} (\mathbf{k}_2\cdot (\mathbf{x}_2-\mathbf{x}_0) + \mathbf{k}_1\cdot (\mathbf{x}_1-\mathbf{x}_0) ) } \operatorname{e}^{-\mathring{\imath} (\omega_2 (t_2 - t_0) + \omega_2 (t_1 - t_0) ) }\nonumber\\ &amp; + n_0(n_0-1)\operatorname{e}^{\mathring{\imath} (\mathbf{k}_2\cdot(\mathbf{x}_2-\mathbf{x}_0) + \mathbf{k}_1\cdot(\mathbf{x}_1-\mathbf{x}_0) ) } \operatorname{e}^{-\mathring{\imath} (\omega_2 (t_2-t_0) + \omega_1 (t_1-t_0) ) } \Big\}. \end{align*}

This concludes the demonstration that the Doi–Peliti framework and Dean’s equation produce identical results for the two-point correlation function. Equation (50) illustrates the central cancellation mechanism, which we generalise to the relevant observables below, in particular appendix A. As equation (38) is a perturbative term, the resulting branching diagrams in equation (53) *discount* contributions due to independent particle movement, shown as two parallel propagators in equation (53), of which there are $n_0^2$ rather than $n_0(n_0-1)$.

Performing the calculation above immediately in direct time and real space is most easily done assuming a particular time ordering, say $t_2 \gt t_1 \gt t_0$. In that case, Doi–Peliti produces

\begin{align*} C(\mathbf{x}_1,\mathbf{x}_2,t_1,t_2)&amp; = n_0 \frac{\mathrm{e}^{-\frac{(\mathbf{x}_2-\mathbf{x}_1)^2}{4D(t_2-t_1)}}}{(4\pi D(t_2-t_1))^{d/2}}\frac{\mathrm{e}^{-\frac{(\mathbf{x}_1-\mathbf{x}_0)^2}{4D(t_1-t_0)}}}{(4\pi D(t_1-t_0))^{d/2}}\nonumber\\ &amp; \quad + n_0(n_0-1) \frac{\mathrm{e}^{-\frac{(\mathbf{x}_1-\mathbf{x}_0)^2}{4D(t_1-t_0)}}}{(4\pi D(t_1-t_0))^{d/2}}\frac{\mathrm{e}^{-\frac{(\mathbf{x}_2-\mathbf{x}_0)^2}{4D(t_2-t_0)}}}{(4\pi D(t_2-t_0))^{d/2}} \end{align*}

directly from equation (42) using the propagator equation (11). As $t_2\gt t_1$, only the first and the last diagrams of equation (42) contribute, the first due to a particle travelling from **x**_0_ to **x**_1_ and then to **x**_2_ and the last due to two particles travelling independently. In the limit of $t_2\downarrow t_1$ the first term, proportional to *n*_0_, becomes $n_0 \delta(\mathbf{x}_2-\mathbf{x}_1) (4\pi D(t_1-t_0))^{-d/2}\operatorname{e}^{-(\mathbf{x}_1-\mathbf{x}_0)^2/(4\pi D (t_1-t_0)}$, vanishing if $\mathbf{x}_1\ne\mathbf{x}_2$ as the same particle cannot be at two different places simultaneously. Figure 1 provides a visual illustration of this property.

Although this approach no longer requires regularisation by the extinction rate *r*, it is somewhat more demanding to perform the calculation of the correlation function within Dean’s equation in direct time and real space using equation (34), because the Dean-vertex requires a convolution over the time and the position where the virtual branching takes place,


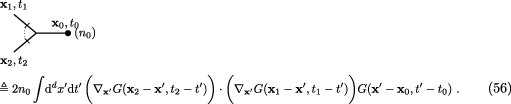


After some algebra, Dean’s equation produces of course the same correlation function equation (55) as Doi–Peliti.

In explicit calculations below, notably appendix A, we will make use of a mixed momentum-time, $k,t$, parameterisation, for which we briefly outline the cancellation mechanism in the following. In Doi–Peliti, the diagrams equation (42) can immediately be written as

\begin{align*} C(\mathbf{x}_1,\mathbf{x}_2, t_1, t_2)&amp; = \int {\mathrm{d}\mkern-8mu^{-}}^d k_2{\mathrm{d}\mkern-8mu^{-}}^d k_1\nonumber\\ &amp;\quad \times\bigg\{ n_0 \operatorname{e}^{\mathring{\imath} (\mathbf{k}_2\cdot(\mathbf{x}_2-\mathbf{x}_1) + \mathbf{k}_1\cdot(\mathbf{x}_1-\mathbf{x}_0) ) } \theta(t_2-t_1)\theta(t_1-t_0) \operatorname{e}^{-D\mathbf{k}_2^2 (t_2-t_1) -D\mathbf{k}_1^2 (t_1-t_0) ) }\nonumber\\ &amp;\quad + n_0 \operatorname{e}^{\mathring{\imath} (\mathbf{k}_2\cdot(\mathbf{x}_2-\mathbf{x}_0) + \mathbf{k}_1\cdot(\mathbf{x}_1-\mathbf{x}_2) ) } \theta(t_1-t_2)\theta(t_2-t_0)\operatorname{e}^{-D\mathbf{k}_1^2(t_1-t_2) -D\mathbf{k}_2^2 (t_2-t_0) ) } \nonumber\\ &amp;\quad + n_0(n_0-1) \operatorname{e}^{\mathring{\imath} (\mathbf{k}_2\cdot(\mathbf{x}_2-\mathbf{x}_0) + \mathbf{k}_1\cdot(\mathbf{x}_1-\mathbf{x}_0) ) } \theta(t_2-t_0)\theta(t_1-t_0)\nonumber\\ &amp;\quad \times \operatorname{e}^{-D\mathbf{k}_2^2(t_2-t_0) -D\mathbf{k}_1^2 (t_1-t_0) ) } \bigg\} \end{align*}

by replacing each of the bare propagators of equation (41) by equation (12) and making use of the *δ*-functions on the momenta, or by direct interpretation of the diagrams.

Dean’s equation, equation (46), on the other hand, produces

\begin{align*} C(\mathbf{x}_1,\mathbf{x}_2, t_1, t_2)&amp; = \int {\mathrm{d}\mkern-8mu^{-}}^d k_2{\mathrm{d}\mkern-8mu^{-}}^d k_1{\mathrm{d}\mkern-8mu^{-}}^d k_0 \operatorname{e}^{\mathring{\imath}\mathbf{k}_2\cdot\mathbf{x}_2} \operatorname{e}^{\mathring{\imath}\mathbf{k}_1\cdot\mathbf{x}_1} \operatorname{e}^{\mathring{\imath}\mathbf{k}_0\cdot\mathbf{x}_0}\delta\mkern-9mu^{-}(\mathbf{k}_2+\mathbf{k}_1+\mathbf{k}_0)\nonumber\\ &amp; \quad \times\Bigg\{ (-2n_0D\mathbf{k}_1\cdot\mathbf{k}_2) \int_{-\infty}^\infty\mathrm{d}t^{^{\prime}}\, \theta(t_2-t^{^{\prime}}) \nonumber\\ &amp;\quad \times \operatorname{e}^{-D\mathbf{k}_2^2(t_2-t^{^{\prime}})} \theta(t_1-t^{^{\prime}}) \operatorname{e}^{-D\mathbf{k}_1^2(t_1-t^{^{\prime}})} \theta(t^{^{\prime}}-t_0) \operatorname{e}^{-D\mathbf{k}_0^2(t^{^{\prime}}-t_0)} \Bigg\}\nonumber\\ &amp; \quad + \int {\mathrm{d}\mkern-8mu^{-}}^d k_2{\mathrm{d}\mkern-8mu^{-}}^d k_1 \operatorname{e}^{\mathring{\imath}\mathbf{k}_2\cdot(\mathbf{x}_2-\mathbf{x}_0)} \operatorname{e}^{\mathring{\imath}\mathbf{k}_1\cdot(\mathbf{x}_1-\mathbf{x}_0)}\nonumber\\ &amp;\quad\times \left\{ n_0^2 \theta(t_2-t_0) \operatorname{e}^{-D\mathbf{k}_2^2(t_2-t_0)} \theta(t_1-t_0) \operatorname{e}^{-D\mathbf{k}_1^2(t_1-t_0)} \right\}\ , \end{align*}

with the convolution over *t* ^ʹ^, the time of the virtual branching in the first diagram. While the lower limit of this integral is fixed to *t*_0_ by $\theta(t^{^{\prime}}-t_0)$, the upper limit is $t_{\textrm{min}} = \min{(t_1,t_2)}$ via the product of two Heaviside *θ*-functions. Its two possible values generate two terms as in equation (42), conditioned by *θ*-functions. Using the *δ*-function in the first line of equation (58) to eliminate the integral over **k**_0_, the *n*_0_ branching terms each produce

\begin{align*} &amp; \int_{t_0}^{t_{\textrm{min}}} \mathrm{d}t^{^{\prime}}\, \operatorname{e}^{-D\mathbf{k}_2^2(t_2-t^{^{\prime}})} \operatorname{e}^{-D\mathbf{k}_1^2(t_1-t^{^{\prime}})} \operatorname{e}^{-D(\mathbf{k}_1+\mathbf{k}_2)^2(t^{^{\prime}}-t_0)} \nonumber \\ &amp; \quad = \operatorname{e}^{-D\mathbf{k}_2^2(t_2-t_0)} \operatorname{e}^{-D\mathbf{k}_1^2(t_1-t_0)} \left(1-\operatorname{e}^{-2D\mathbf{k}_1\cdot\mathbf{k}_2(t_{\textrm{min}}-t_0)}\right) \frac{1} {2D\mathbf{k}_1\cdot\mathbf{k}_2} \ . \end{align*}

The 1-term in the bracket is independent of *t*_min_ and cancels with *n*_0_ of the $n_0^2$ disconnected terms. The remaining terms can be simplified using for example

\begin{align*} \operatorname{e}^{-D\mathbf{k}_1^2(t_1-t_0)} \operatorname{e}^{-2D\mathbf{k}_1\cdot\mathbf{k}_2(t_1-t_0)} = \operatorname{e}^{-D(\mathbf{k}_1+\mathbf{k}_2)^2(t_1-t_0)} \operatorname{e}^{D\mathbf{k}_2^2(t_1-t_0)} \end{align*}

in the case of $t_{\textrm{min}} = t_1$ and, after a shift in **k**_*i*_, such as $\mathbf{k}_1+\mathbf{k}_2\to\mathbf{k}_1$ in the example above, reproduce the result from Doi–Peliti, equation (57). This concludes the illustration.

To summarise this section, the correlation function of the particle position of *n*_0_ non-interacting particles is not a single term, as it needs to capture multiple scenarios of particles moving, while keeping track of the particle nature of the constituent degrees of freedom. Both frameworks result in the same expressions, such equations (45), (54), (55) and (57). A cancellation mechanism such as equation (49) in the $\mathbf{k},\omega$ parameterisation and the convolution in equation (59) for $\mathbf{k},t$, connects Doi–Peliti and Dean, revealing that the perturbative, virtual branching in Dean’s framework is in fact a sum of sequential propagation of a single particle and independent propagation of two distinct ones.

The calculation in this preliminary section suggests that the interaction vertex equation (38) in Dean’s formalism, a vertex that would be missing in a naive response field theory of the corresponding Fokker–Planck equation, contains the same information as the commutation relation of the Doi–Peliti ladder operators. The importance of this observation will become evident in sections 4 and 5, where we analyse the particle nature in greater detail.

## 3. Probing for particle entity

Within the Dean framework $\rho(\mathbf{x},t)$ denotes the instantaneous particle number density in state **x** at time *t*. We define particle entity as a property of the evolution equation for $\rho(\mathbf{x},t)$ whereby this time-dependent random variable can be written as a finite sum of ‘single particle densities’ with integer coefficients. In the case of a discrete phase space, the single-particle density for a particle in state $\bar{\mathbf{x}}$ is the Kronecker-delta with unit prefactor, $\delta_{\mathbf{x},\bar{\mathbf{x}}}$. For continuous degrees of freedom, the single-particle density for a particle in state $\bar{\mathbf{x}}$ is the Dirac-delta distribution normalised to unity, $\delta(\mathbf{x}-\bar{\mathbf{x}})$. Correspondingly,

\begin{align*} \rho(\mathbf{x},t) = \begin{cases} \sum_{i} n_i(t) \delta_{\mathbf{x},\bar{\mathbf{x}}_i}, &amp; \textrm{for discrete states} \\ \sum_{i} n_i(t) \delta(\mathbf{x}-\bar{\mathbf{x}}_i), &amp; \textrm{for continuous states} \end{cases} \end{align*}

where $n_i(t) \in \mathbb{N}$. It follows from this requirement that the integral of the particle number density $\rho(\mathbf{x},t)$ over any (sub-)volume Ω of the space is an integer-valued random variable,

\begin{align*} \forall \Omega \subset \mathbb{R}^d: \begin{cases} \sum_{i \in \Omega} \rho(\mathbf{x}_i,t) \in \mathbb{N}, &amp; \textrm{for discrete states} \\ \int_\Omega d^dx \rho(\mathbf{x},t) \in \mathbb{N}, &amp; \textrm{for continuous states} \end{cases}, \end{align*}

where integrals are performed in the Dirac measure, rather than by expressing the Dirac-delta distribution as the limit of a series of bump functions under a Lebesgue integral.

For discrete states, equation (62) also implies equation (61), i.e. there can be no densities satisfying equation (62) that are not a sum of Kronecker deltas with integer coefficient. We leave the proof that this equivalence also holds in the continuum for future work. Such a proof will surely draw on the arbitrariness of Ω, which can be used to include or exclude from the integral in equation (62) any part of $\rho(\mathbf{x},t)$. In the case of stochastic dynamics, it is convenient to re-express the condition equation (62) in terms of an expectation value as

\begin{align*} \left\langle \mathrm{exp}\left( 2\pi \mathring{\imath} \int_\Omega \mathrm{d}^{d}x \rho(\mathbf{x},t) \right) \right\rangle = 1 ~, \end{align*}

which needs to be satisfied for any volume Ω and all times *t*. Equation (63) will play the role of a signature of particle entity in the following.

Obviously, equation (62) implies equation (63). Yet, expressing the particle entity condition as an expectation might appear less stringent than demanding it at the level of individual trajectories. However, rewriting equation (63) as $\langle \cos(2\pi \int \mathrm{d}^{d}x \rho(\mathbf{x}, t))\rangle = 1$ on the basis of $\rho(\mathbf{x},t)$ being real, shows that the integral must be integer valued almost surely, because $\mathbb{R} \ni \cos(x) \leqslant 1$ for $x\in\mathbb{R}$.

In order to ease the calculation of the left-hand side of equation (63) for a particular field theory of interest, we can expand the complex exponential as a Taylor series and invoke linearity of the expectation to obtain the particle entity signature,


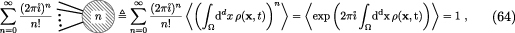


where the left-hand side is now a function of the *n*th full moment of the integrated particle number density, $\left\langle \left( \int_\Omega \mathrm{d}^{d}x \rho(\mathbf{x},t) \right)^n \right\rangle$. In equation (64) we have also introduced the diagrammatic notation for the *n*th full moment of the *integrated* particle number density. The hatched vertex henceforth indicates generally a sum of possibly disconnected diagrams involving an arbitrary amount of sources. More specifically, here it is the *n*-fold spatial integral of a sum of products of connected diagrams. An example for such a sum is equation (46). These moments are perfectly suited for being calculated in both Doi–Peliti and response field theories, as done in the following.

An alternative form of our particle entity signature can be obtained by recognising that the left hand side of equation (63) is also the moment-generating function $\langle \operatorname{e}^{z X}\rangle$ of the random variable $X = \int_\Omega \mathrm{d}\mathbf{x}\, \rho(\mathbf{x},t)$ evaluated at $z = 2 \pi \mathring{\imath}$. It is a well-known result from the field-theoretic literature on equilibrium critical phenomena [9, 25] that the generating function of the full moments $\langle X^n \rangle$ can be expressed as the exponential of the generating function of the so-called connected moments, denoted $\langle X^n \rangle_c$. Outside the field-theoretic literature, connected moments are usually referred to as cumulants [26]. While one would normally expect source fields $j(\mathbf{x},t)$ to be introduced corresponding to a conjugate variable *z* at each point in space and time, in the present context a single variable suffices, as in equation (64) every field $\rho(\mathbf{x},t)$ is integrated over the same volume Ω. Diagrammatically,


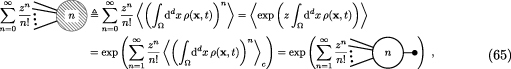


where we have introduced the notation for the *n*th *connected* moment of the integrated particle number density, shown as a circular vertex on the right, which differs from that of the corresponding full moment also by the presence of an explicit, single, ingoing propagator emerging from a single source, shown as a filled circle, the only possible form of a connected diagram contributing to moments of the density. In equilibrium statistical mechanics, this relationship provides the connection between the partition function and the Helmholtz free energy [9, 25]. Generating functions of observables such as *n*-point correlation functions of $\rho(\mathbf{x},t)$ can be reduced to those of connected diagrams as long as the observables can be written as (functional) derivatives of an exponential and provided that each resulting diagram can be written as a product of connected diagrams. Under these conditions, equation (65) does all the right accounting.

Evaluating equation (65) at $z = 2\pi\mathring{\imath}$, according to equation (64) one can write

\begin{align*} \mathrm{exp}\left( \sum_{n=1}^\infty \frac{(2\pi \mathring{\imath})^n}{n!} \left\langle \left( \int_\Omega \mathrm{d}^{d}x \rho(\mathbf{x},t) \right)^n \right\rangle_c \right) = 1 \ , \end{align*}

or, equivalently,


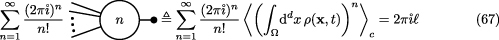


for some integer $\ell \in \mathbb{Z}$, on the basis of the connected moments of the particle number, to be compared to the particle signature on the basis of the full moments, equation (64).

## 4. Particle entity in Doi–Peliti

Doi–Peliti field theories are designed to respect particle entity and they do indeed do so on a rather fundamental level. To probe for particle entity, we want to use equation (63) with $\rho(\mathbf{x},t)$ replaced by an object suitable for a Doi–Peliti field theory. In such a field theory, the instantaneous particle number at any position **x** is probed by the number operator $\hat{n}(\mathbf{x}) = a^\dagger(\mathbf{x})a(\mathbf{x})$. The expected particle number at position **x** and time *t* is therefore

\begin{align*} \langle n(\mathbf{x},t) \rangle = \left\langle \unicode{x263C} \right| a^\dagger(\mathbf{x})a(\mathbf{x}) \left| \Psi (t) \right\rangle \ , \end{align*}

using a continuum version of the notation introduced in section 2, in particular equation (3). While this expectation might be any non-negative real, the instantaneous $a^\dagger(\mathbf{x})a(\mathbf{x})$ is an integer. As already discussed in section 3, equation (63), we therefore expect

\begin{align*} \left\langle \unicode{x263C} \right| \operatorname{exp}\left(2\pi\mathring{\imath} a^\dagger(\mathbf{x})a(\mathbf{x})\right) \left| \Psi (t) \right\rangle = 1 \end{align*}

to hold for every **x**, as $\operatorname{e}^{2\pi\mathring{\imath} n} = 1$ for any $n\in\mathbb{Z}$. If this holds for every point **x**, it also holds for every patch Ω, since

\begin{align*} \left\langle \unicode{x263C} \right| \operatorname{exp}\left(2\pi\mathring{\imath} \sum_{\mathbf{x} \in \Omega} a^\dagger(\mathbf{x})a(\mathbf{x})\right) \left| \Psi (t) \right\rangle = \left\langle \unicode{x263C} \right| \prod_{\mathbf{x} \in \Omega} \operatorname{exp}\left(2\pi\mathring{\imath} a^\dagger(\mathbf{x})a(\mathbf{x})\right) \left| \Psi (t) \right\rangle ~, \end{align*}

where we have used that operators at different **x** commute. In the continuum, one might argue that the particle number at **x** can only ever be 0 or 1, possibly leading to some simplifications, but on the lattice occupation is not bound to be sparse in this sense.

To show that equation (69) is indeed satisfied in general Doi–Peliti field theories, we recall that the state $\left| \Psi (t) \right\rangle$ can be written as a statistical superposition of integer occupation number states on the lattice, equation (3), which are eigenstates of the number operator $a^\dagger({\mathbf{x}})a({\mathbf{x}})$ with integer eigenvalue $n_{\mathbf{x}} \in \mathbb{N}$. The left hand side of equation (69) thus immediately simplifies to the desired result,

\begin{align*} \sum_{\{n_i\}} P(\{n_i\},t) \operatorname{exp}\left(2\pi\mathring{\imath} n_{\mathbf{x}}\right) \left\langle \unicode{x263C} \right| \left \{n_i\} \right\rangle = \sum_{\{n_i\}} P(\{n_i\},t) = 1~. \end{align*}

To see more clearly which features of the formalism are responsible for this particular property of the state $\left| \Psi (t) \right\rangle$ being preserved under the dynamics, and to see this at the level of fields, we can alternatively follow the standard procedure, outlined in equation (15), to express the operator $\operatorname{exp}\left(2\pi\mathring{\imath} a^\dagger(\mathbf{x})a(\mathbf{x})\right)$ in terms of scalar fields $\psi(\mathbf{x},t)$ and $\psi^\dagger(\mathbf{x},t)$. The simple mapping of operator to field applies as soon as the operators are normal ordered,

\begin{align*} \operatorname{exp}\left(z a^\dagger(\mathbf{x})a(\mathbf{x})\right) &amp;= \sum_{n=0}^\infty \frac{1}{n!} z^n \left(a^\dagger(\mathbf{x})a(\mathbf{x})\right)^n \end{align*}

\begin{align*} &amp; \qquad \qquad \qquad \qquad =\sum_{n=0}^\infty \frac{1}{n!} z^n \sum_{k=0}^n \left\{ \begin{array}{c} n \\ k \end{array} \right\} \left( a^{\dagger}(\mathbf{x})\right)^k a(\mathbf{x})^k \end{align*}

where we have replaced $2\pi\mathring{\imath}$ by *z* to improve readability and used [23] to normal order $\left(a^\dagger(\mathbf{x})a(\mathbf{x})\right)^n$. In terms of fields, the observable equation (70) is thus

\begin{align*} &amp; \;\mathcal{O}= \left\langle \unicode{x263C} \right| \operatorname{exp}\left(z \sum_{x\in \Omega} \left(a^{\dagger}(\mathbf{x})\right) a(\mathbf{x})\right) \left| \Psi(\mathbf{x},t) \right\rangle \end{align*}

\begin{align*} &amp; \quad =\left\langle \unicode{x263C} \right| \prod_{x\in \Omega} \sum_{n=0}^{\infty} \frac{1}{n!} z^n \sum_{k=0}^{n} \left\{ \begin{array}{c} n \\ k \end{array} \right\} \left(a^{\dagger}(\mathbf{x})\right)^k a(\mathbf{x})^k \left| \Psi(\mathbf{x},t) \right\rangle \end{align*}

\begin{align*} &amp; \quad ={\left\langle \prod_{x\in \Omega} \sum_{n=0}^{\infty} \frac{1}{n!} z^n \sum_{k=0}^{n} \left\{ \begin{array}{c} n \\ k \end{array} \right\} \psi^k(\mathbf{x},t)\, \mathbb{I}(\tilde{\psi}(0) + 1) \right\rangle} \end{align*}

\begin{align*} &amp; \quad ={\left\langle \operatorname{exp}\left(\sum_{x\in \Omega}\psi(\mathbf{x},t) (e^z-1)\right)\mathbb{I}(\tilde{\psi}(0) + 1) \right\rangle} \end{align*}

as $\left\langle \unicode{x263C} \right| (a^{\dagger}(\mathbf{x}))^k = \left\langle \unicode{x263C} \right|$ [13] and using equation (17) for the initialisation $\mathbb{I}(\tilde{\psi}(0) + 1)$. From equation (76) to (77), we draw on the mixed bivariate generating function for the Stirling numbers of the second kind [27],

\begin{align*} \sum_{n=0}^\infty \sum_{k=0}^n \left\{ \begin{array}{c} n \\ k \end{array} \right\} \frac{1}{n!} z^n y^k = \operatorname{exp}\left(y(\operatorname{e}^{z}-1)\right) \ . \end{align*}

For $z = 2\pi\mathring{\imath}$, and any integer multiple thereof, equation (77) indeed produces $\mathcal{O} = {\langle \mathbb{I}(\tilde{\psi}(0) + 1) \rangle} = 1$ as required by equation (63). Because this calculation never draws on any particular action, but rather on the fundamentals of normal ordering, Doi–Peliti field theories respect particle entity universally in the presence of any interactions and potentials.

## 5. Particle entity in response field theories: Dean’s equation

Demonstrating that the response field theory derived from Dean’s equation possesses particle entity turns out to be a much more challenging task, which requires us to compute explicitly the connected moments of the integrated particle number density to arbitrary order. This calculation draws on the specific action equations (23) and (24) as we perform it here for the case of non-interacting diffusive particles without external potential. This is done most conveniently by first computing the connected moments of the density in the mixed momentum-time representation, where Dean’s action reads

\begin{align*} A[\rho,\tilde{\rho}] &amp;= \int {\mathrm{d}\mkern-8mu^{-}}^d k \mathrm{d}t\, \tilde{\rho}(\mathbf{k},t) (\partial_t + D \mathbf{k}^2) \rho(-\mathbf{k},t) - \tilde{\rho}(\mathbf{k},t) \sum_i n_i \mathrm{e}^{\mathring{\imath} \mathbf{k} \cdot \mathbf{x}_i} \delta(t-t_i) \nonumber \\ &amp;\quad + \int {\mathrm{d}\mkern-8mu^{-}}^d k {\mathrm{d}\mkern-8mu^{-}}^d k^{^{\prime}} \mathrm{d}t\, D (\mathbf{k} \cdot \mathbf{k}^{^{\prime}}) \tilde{\rho}(\mathbf{k},t) \tilde{\rho}(\mathbf{k}^{^{\prime}},t) \rho(-(\mathbf{k}+\mathbf{k}^{^{\prime}}),t). \end{align*}

In this parametrisation, we find (appendix A, equation (A26))

\begin{align*} \langle \rho(\mathbf{k}_1,t)\ldots\rho(\mathbf{k}_n,t)\rangle_c&amp; = n_0 \theta(t-t_0)\operatorname{e}^{\mathring{\imath}\mathbf{k}_1+\ldots\mathbf{k}_n\cdot\mathbf{x}_0} \sum_{m=1}^{n} (-1)^{m-1} (m-1)!\nonumber\\ &amp;\quad \times \sum_{\{\mathbb{P}_1,\dots,\mathbb{P}_m\}\in \mathcal{P}\left(\{1,\dots,n\},m\right)} \operatorname{e}^{-T(t-t_0) \sum_{i=1}^m \mathbf{K} \left(\mathbb{P}_i\right)^2} \end{align*}

with $\mathcal{P}\left(\{1,\dots,n\},m\right)$ the set of all partitions of the set $\{1,\dots,n\}$ into *m* non-empty, distinct subsets $\mathbb{P}_i$ with $i = 1,\ldots,m$, i.e. $\cup_{i = 1}^m \mathbb{P}_i = \{1,2,\dots,n\}$ and $\mathbb{P}_i \cap \mathbb{P}_j = \emptyset$ for $i\ne j$. The sum thus runs over all possible partitions of {1,2,…,*n*} into *m* non-empty sets. There is one partition for *m* = *n* and *n* for *m* = 1. The vector featuring in the right-most exponential of equation (80)

\begin{align*} \mathbf{K} \left(\mathbb{P}_i\right) = \sum_{p\in\mathbb{P}_i} \mathbf{k}_p \end{align*}

is the total momentum given by the indices in the subset $\mathbb{P}_i$, i.e. it is the total momentum of the subset $\mathbb{P}_i$, and by linearity, $\mathbf{K} \left(\mathbb{A}\right)+\mathbf{K} \left(\mathbb{B}\right) = \mathbf{K} \left(\mathbb{A}\cup\mathbb{B}\right)$. For example, one partition into two of $\{1,2,3,4\}$ is $\{\mathbb{P}_1 = \{1,2,4\},\mathbb{P}_2 = \{3\}\}$, which is one of seven elements of $\mathcal{P}\left(\{1,2,3,4\},2\right)$. In this example, the momenta of the subsets are $\mathbf{K} \left(\mathbb{P}_1\right) = \mathbf{k}_1+\mathbf{k}_2+\mathbf{k}_4$ and $\mathbf{K} \left(\mathbb{P}_2\right) = \mathbf{k}_3$. Diagrammatically, the right-hand side of equation (80) is obtained by summing over all connected, topologically distinct diagrams with a single incoming propagator and *n* outgoing propagators labelled by the external momenta **k**_*i*_ ($i = 1,\ldots,n$), where we need to account for all non-equivalent permutations of the latter.

The connected moments of the integrated particle number density in a patch Ω are then obtained by Fourier back-transforming equation (80) into position-time representation and integrating over the probing locations $\mathbf{x}_i \in \Omega$,

\begin{align*} &amp;\left\langle \int_\Omega \mathrm{d}^{d}{x_1}\ldots\ \mathrm{d}^{d}{x_n} \rho(\mathbf{x}_1,t)\ldots\rho(\mathbf{x}_n,t)\right\rangle_c \end{align*}

\begin{align*} &amp; \quad= \int_\Omega \prod_{i=1}^n \mathrm{d}^{d}{x_i} \int \prod_{j=0}^n {\mathrm{d}\mkern-8mu^{-}}^d k_j \operatorname{exp}\left(-\mathring{\imath} \sum_{\ell=1}^n \mathbf{k}_\ell\cdot\mathbf{x}_\ell \right)\langle \rho(\mathbf{k}_1,t)\ldots\rho(\mathbf{k}_n,t)\rangle_c \end{align*}

\begin{align*} &amp; \quad =\int_\Omega \prod_{i=1}^n \mathrm{d}^{d}{x_i} \int \prod_{j=1}^n {\mathrm{d}\mkern-8mu^{-}}^d k_j \operatorname{exp}\left(-\mathring{\imath} \sum_{\ell=1}^n \mathbf{k}_\ell\cdot(\mathbf{x}_\ell-\mathbf{x}_0) \right)\nonumber\\ &amp; \qquad \times n_0 \theta(t-t_0) \sum_{m=1}^{n} (-1)^{m-1} (m-1)! \sum_{\{\mathbb{P}_1,\dots,\mathbb{P}_m\}\in \mathcal{P}\left(\{1,\dots,n\},m\right)} \operatorname{e}^{-D(t-t_0) \sum_{p=1}^m \mathbf{K} \left(\mathbb{P}_p\right)^2}. \end{align*}

The integrals in equation (84) can be carried out partition by partition, by taking the integration inside the summation over $m = 1,\dots,n$ and the partitions $\{\mathbb{P}_1,\dots\}\in \mathcal{P}\left(\{1,\dots,n\},m\right)$. As **x**_*i*_ and **k**_*j*_ are both dummy variables, we may think of subset $\mathbb{P}_p$ containing indices $1,\dots,a$ with $a = |\mathbb{P}_p|$, so that the integrals to be carried out for each $p = 1,\dots,m$ are

\begin{align*} J_p= \int_\Omega \prod_{i=1}^a \mathrm{d}^{d}{x_i} \int \prod_{j=1}^a {\mathrm{d}\mkern-8mu^{-}}^d k_j \operatorname{exp}\left(-\mathring{\imath} \sum_{\ell=1}^a \mathbf{k}_\ell\cdot(\mathbf{x}_\ell-\mathbf{x}_0) \right) \operatorname{e}^{-D(t-t_0) \mathbf{K} \left(\mathbb{P}_p\right)^2}~. \end{align*}

In this indexing we have $\mathbf{K} \left(\mathbb{P}_p\right) = \mathbf{k}_1+\dots+\mathbf{k}_a$ which simplifies to $\tilde{\mathbf{k}}_1$ after suitable shifting of the origin in the integration over $\mathbf{k}_1 = \tilde{\mathbf{k}}_1-(\mathbf{k}_2+\dots+\mathbf{k}_a)$, so that

\begin{align*} J_p= \int_\Omega \prod_{i=1}^a \mathrm{d}^{d}{x_i} \int {\mathrm{d}\mkern-8mu^{-}}^d \tilde{k}_1 \operatorname{e}^{-\mathring{\imath} \tilde{\mathbf{k}}_1\cdot(\mathbf{x}_1-\mathbf{x}_0) } \operatorname{e}^{-D(t-t_0) \tilde{\mathbf{k}}_1^2} \times \int \prod_{j=2}^a {\mathrm{d}\mkern-8mu^{-}}^d k_j \operatorname{exp}\left(-\mathring{\imath} \sum_{\ell=2}^a \mathbf{k}_\ell\cdot(\mathbf{x}_\ell-\mathbf{x}_1) \right)\nonumber\\ \end{align*}

\begin{align*} \quad &amp;= \int_\Omega \prod_{i=1}^a \mathrm{d}^{d}{x_i} G(\mathbf{x}_1 - \mathbf{x}_0,t-t_0) \delta(\mathbf{x}_2-\mathbf{x}_1) \ldots \delta(\mathbf{x}_a-\mathbf{x}_1) \end{align*}

\begin{align*} \quad &amp;= I_\Omega(t-t_0) \end{align*}

where we have used equations (34) and (36) in equation (86*b*) and introduced

\begin{align*} I_\Omega(t-t_0)=\int_{\Omega} \mathrm{d}^{d}x G(\mathbf{x} - \mathbf{x}_0,t-t_0) ~, \end{align*}

in equation (86*c*), which is the probability to find a particle at time *t* within the volume Ω that had at time *t*_0_ been placed at **x**_0_. We may drop the time-dependence of $I_\Omega$ where that improves readability.

As *J*_*p*_ is independent of the specific partition, the sum over partitions $\{\mathbb{P}_1,\dots,\mathbb{P}_m\}\in\mathcal{P}\left(\{1,\dots,n\},m\right)$ in equation (84) amounts to multiplying a product of *m* such integrals by the number of partitions, given by the Stirling numbers of the second kind. Overall,


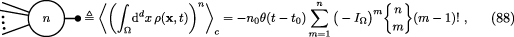


which provides us with the information we need to probe the theory for particle entity. It is a key result of the present work. Its derivation is generalised to distinct initial positions in appendix B.

Using the particle entity signature based on connected diagrams, equation (67), confronts us with some undesirable hurdles due to convergence. We therefore turn our attention to the full moments, which can be constructed from the connected moments via equation (65), so that for $t\gt t_0$,


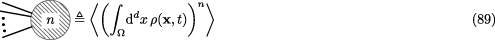

\begin{align*} \;\;\, &amp;= \left. \frac{\mathrm{d}^n}{\mathrm{d}z^n} \right|_{z=0} \operatorname{exp}\left( \sum_{m=1}^\infty \frac{z^m}{m!} \left\langle\left( \int_\Omega \mathrm{d}^{d}x \rho(\mathbf{x},t)\right)^m\right\rangle_c \right) \end{align*}

\begin{align*} \;\;\, &amp;= \left. \frac{\mathrm{d}^n}{\mathrm{d}z^n} \right|_{z=0} \operatorname{exp}\left(\sum_{m=1}^\infty \frac{z^m}{m!} (-n_0) \sum_{\ell=1}^{m} (-I_\Omega)^\ell \left\{ \begin{array}{c} m \\ \ell \end{array} \right\} (\ell-1)! \right) \end{align*}

\begin{align*} \;\;\, &amp;= \left. \frac{\mathrm{d}^n}{\mathrm{d}z^n} \right|_{z=0} \operatorname{exp}\left(-n_0 \sum_{\ell=1}^{\infty} (-I_\Omega)^\ell (\ell-1)! \sum_{m=\ell}^\infty \frac{z^m}{m!} \left\{ \begin{array}{c} m \\ \ell \end{array} \right\}\right) \, , \end{align*}

where we have changed the order of summation in the exponential to arrive at the final equality. This step deserves further scrutiny below. Using in equation (92) the generating function of the Stirling numbers [27] in the form

\begin{align*} \sum_{m=\ell}^\infty \frac{z^m}{m!} \left\{ \begin{array}{c} m \\ \ell \end{array} \right\} = \frac{(e^z - 1)^\ell}{\ell!}~, \end{align*}

as used previously in the Doi–Peliti field theory, equation (78), leads to


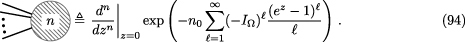


We briefly return to the change of the order of summation from equation (91) to (92). To justify this, we require *absolute* convergence

\begin{align*} \sum_{\ell=1}^\infty \sum_{m=\ell}^\infty \frac{|z|^m}{m!} I_\Omega^\ell \left\{ \begin{array}{c} m \\ \ell \end{array} \right\} (\ell-1)!\lt \infty \end{align*}

for $I_\Omega\in[0,1]$ and *z* within a finite vicinity around the origin, given the repeated differentiation in equation (94). As equation (93) holds for all $z\in\mathbb{C}$, we require $\sum_{\ell = 1}^\infty I_\Omega^\ell (\operatorname{exp}\left(|z|\right)-1)^\ell/\ell\lt\infty$ and thus $|z|\lt\ln(2)$ by the ratio test.

Rewriting the exponent in equation (94) for any $|z|\lt\ln(2)$ as a logarithm,

\begin{align*} \sum_{l=1}^\infty \frac{(-x)^l}{l} = - \log(1+x) \, , \end{align*}

we have


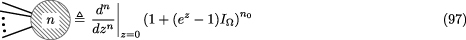

\begin{align*} \qquad\quad\! &amp; = \left. \frac{\mathrm{d}^n}{\mathrm{d}z^n} \right|_{z=0} \sum_{k=0}^{n_0} \binom{n_0}{k} (\mathrm{e}^z-1)^k I_\Omega^k \end{align*}

\begin{align*} \qquad\quad\! &amp;= \sum_{k=0}^{n_0} \binom{n_0}{k} I_\Omega^k \left. \frac{\mathrm{d}^n}{\mathrm{d}z^n} \right|_{z=0} \sum_{j = 0}^k \binom{k}{j} (-1)^j \mathrm{e}^{z(k-j)} \end{align*}

\begin{align*} \qquad\quad\! &amp;= \sum_{k=0}^{n_0} \binom{n_0}{k} I_\Omega^k \sum_{j = 0}^k \binom{k}{j} (-1)^j(k-j)^n \, , \end{align*}

and using the definition of the Stirling numbers of the second kind [27]

\begin{align*} \sum_{g=0}^k {k \choose g} (-1)^g (k-g)^n = k! \left\{ \begin{array}{c} n \\ k \end{array} \right\} \, , \end{align*}

we finally arrive at


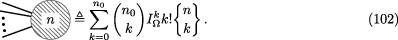


This is the first central result of the present section. The derivation above implicitly used that *n*_0_ is integer. This requirement turns out to also be important for the physical interpretation of the result, which we discuss in detail in appendix C.

We now derive a different form of equation (102), which offers a more immediate physical interpretation. To this end, we draw on the identity

\begin{align*} \sum_{k=0}^{n_0} {n_0 \choose k} (1-x)^{n_0-k} x^k k^n = \sum_{\ell=0}^{n_0} {n_0\choose \ell} x^\ell \ell! \left\{ \begin{array}{c} n \\ \ell \end{array} \right\} \end{align*}

which can be obtained by resolving the binomial $(1-x)^{n_0-k}$ on the left into a sum, collecting terms of order $x^{\ell}$ and then using ${n_0 \choose k}{n_0-k \choose \ell-k} = {n_0 \choose \ell}{\ell \choose k}$ to arrive at an expression that simplifies to the final sum by means of equation (101).

Using equation (103) in equation (102) gives

\begin{align*} {\left\langle \left( \int_\Omega \mathrm{d}^{d}x \rho(\mathbf{x},t)\right)^n \right\rangle} = \sum_{k=0}^{n_0}{n_0 \choose k} \bigg(1-I_\Omega(t-t_0)\bigg)^{n_0-k} \bigg(I_\Omega(t-t_0) \bigg)^k k^n \ , \end{align*}

which has a rather simple physical interpretation: of the (integer) *n*_0_ particles, *k* can be found in the volume Ω, each independently with probability $I_\Omega$, so that the probability of such a configuration is that of a repeated Bernoulli trial, ${n_0 \choose k} (1-I_\Omega)^{n_0-k} I_\Omega^k$. As *k* particles are in the relevant volume, the contribution to the *n*th particle number moment is *k* ^*n*^.

In the form equation (104), the particle number moments can be used in our particle entity signature equation (64),


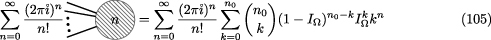

\begin{align*} \qquad \qquad \qquad = \sum_{k=0}^{n_0} \binom{n_0}{k } (1-I_\Omega)^{n_0-k}I_\Omega^k \left( \sum_{n=0}^\infty \frac{(2\pi\mathring{\imath})^n}{n!} k^n \right) = 1~, \end{align*}

where we have used $\mathrm{e}^{2\pi\mathring{\imath} k} = 1$ for $k \in \mathbb{Z}$ in the last bracket and the normalisation of a binomial distribution. To change the order of summation going from equation (105) to (106) we draw on the absolute convergence of the last line equation (106). This concludes the proof of particle entity on the basis of equation (64) in the Dean formalism, the second central result of this section. As already hinted at in section 2.3, we have seen that the precise form of Dean’s vertex equation (38) plays a fundamental role in generating the diagrammatic structure responsible for the enforcement of particle entity in its field theory. We expect that perturbative treatments affecting the vertex form, the extreme case being its complete removal and subsequent treatment of Dean’s equation as a classical diffusion equation, results in the breakdown of particle entity under the dynamics (figure 1).

Therefore a counterexample that fails to satisfy equation (63) would be a regular Fokker–Planck equation without the multiplicative noise of Dean’s equation. The solution is the probability density of a single degree of freedom, but may be misinterpreted as an instantaneous particle density, and powers of this density as correlation functions, thereby allowing a single particle to have a finite density at multiple points simultaneously. Treated in the response-field theory framework this Fokker–Planck equation would lack the three-point vertex of Dean’s theory, meaning that such a naive Fokker–Planck theory would not preserve particle entity.

## 6. Conclusion

To the best of our knowledge, this paper presents the first formalisation and systematic study of the concept of particle entity in the context of statistical field theory. Focusing on two well-known field theoretic formalisms applied to the study of stochastic processes, namely the Doi–Peliti [11] and the Martin–Siggia–Rose–Janssen–De Dominicis [14–16] response field theories, we have demonstrated that particle entity is enforced in a formalism-specific way. In Doi–Peliti field theories, particle entity is built into its foundation, namely in the commutation relation of the ladder operators, equation (1). In the response field theory derived from Dean’s equation, particle entity is a perturbative feature that relies on the precise form of the interaction vertex, equation (38). This ‘Dean vertex’ originates from the Itô-multiplicative noise term in the original Langevin equation (19). It compensates for some overcounting that occurs in the bilinear part of the field theory equation (28), a mechanism that was already identified in an earlier work on the statistics of the non-interacting Brownian gas [20]. As a result, one is faced with more complicated branching diagrams in the response field formalism equipped with particle entity via Dean’s equation compared to the Doi–Peliti formalism, cf equations (40) and (46) or equations (55) and (56).

To test for particle entity, we introduced the condition equation (63), that we rewrote in terms of particle number moments, equation (64), and, on the basis of the identity equation (65), in terms of connected moments, equation (67).

In section 4, we were able to show in a few lines that particle entity according to equation (64) generally holds in Doi–Peliti field theories, equation (77). This finding is independent of the specifics of the action. To demonstrate particle entity for non-interacting, diffusive field theories on the basis of Dean’s equation, we used in section 5 our key result on the connected particle number moments, equation (88), before constructing the main result equation (106) on the basis of the full moments, with some of the more cumbersome calculations relegated to appendix A.

It is interesting to speculate whether our derivation simplifies further by exploiting the well-known identity [9] relating the Legendre transform of the generating function of the connected moments and the effective action, which only depends on the one-particle irreducible (1PI) diagrams. Since 1PIs represent a relatively small subset of all connected diagrams, a particle entity signature of this type might be more easily applicable to theories involving pair interactions, which are beyond the scope of the present analysis.

## Data Availability

No new data were created or analysed in this study.

## Appendix A Induction over connected diagrams

We want to prove that the connected moments of the particle number density in the response field theory derived from Dean’s equation obey equation (80) to all orders of *n*, restated here for convenience:

\begin{align*} \langle \rho(\mathbf{k}_1,t)\ldots\rho(\mathbf{k}_n,t)\rangle_c&amp; = n_0 \theta(t-t_0) \operatorname{e}^{\mathring{\imath}\mathbf{k}_1+\ldots\mathbf{k}_n\cdot\mathbf{x}_0}\sum_{m=1}^{n} (-1)^{m-1} (m-1)\nonumber \\[6pt] &amp;\quad \times \sum_{\{\mathbb{P}_1,\dots,\mathbb{P}_m\}\in \mathcal{P}\left(\{1,\dots,n\},m\right)} \operatorname{e}^{-D(t-t_0) \sum_{i=1}^m \mathbf{K} \left(\mathbb{P}_i\right)^2}. \end{align*}

For this we have to consider all diagrams with a single incoming leg and an arbitrary number *n* of outgoing legs, the first four orders of which are depicted in equations (A2)–(A5)

The shading of the circular vertices above is meant as a visual reminder that we are now dealing with connected moments of the local number *density*, which depend on an unordered set of *n* external momenta $\mathbf{k}_1,\ldots,\mathbf{k}_n$, as opposed to connected moments of the *integrated* number density in a patch Ω (cf equations (80) and (84)). The shaded diagrams are by construction invariant under permutations of the momenta $\mathbf{k}_1,\ldots,\mathbf{k}_n$.

We proceed by determining some of the shaded diagrams. The trivial case, *n* = 1 shown in equation (A2), is given by the propagator equation (36) and the perturbative contribution from the source equation (24) with coupling *n*_0_. Through direct computation in the mixed momentum-time representation, see equation (79), we find for the *n* = 2 case equation (A3):

\begin{align*} = n_0 \operatorname{e}^{\mathring{\imath}\mathbf{k}_1+\mathbf{k}_2\cdot\mathbf{x}_0} \left[ \operatorname{e}^{-D(t-t_0)(\mathbf{k}_1+\mathbf{k}_2)^2}-\operatorname{e}^{-D(t-t_0)(\mathbf{k}_1^2+\mathbf{k}_2^2)} \right] ~, \end{align*}

where the integral over *t* arises from representing the Dean vertex equation (38) in k-t-space. Each Dean vertex comes with a symmetry factor of 2. A factor of $(-2\mathbf{k}_1 \cdot \mathbf{k}_2)^{-1}$ that arises in the *t* integration precisely cancels with the vertex prefactor $-2D \mathbf{k}_1 \cdot \mathbf{k}_2$, which simplifies the result equation (A7) considerably. A similar calculation for the *n* = 3 case equation (A4) yields

The left-hand side of equation (A8) contains the sum over all distinct ways to assign the external momenta to the outgoing legs, as shown in equation (A4). The whole sum of the terms is necessary for the coefficients of the Dean vertices to cancel with the *k*-dependent factor coming down from the *t*-integrations. We will see that this mechanism is instrumental in performing the induction later on.

Based on equations (A7) and (A8) we conjecture and indeed show below that a general connected moment has the form equation (80),

with $\mathcal{P}\left(\{1,\dots,n\},m\right)$ the set of all partitions of the set $\{1,\dots,n\}$ into *m* non-empty, distinct subsets $\mathbb{P}_i$ with $i = 1,2,\dots,m$ so that $\cup_{i = 1}^m \mathbb{P}_i = \{1,2,\dots,n\}$, as introduced after equation (80). The second sum $\sum_{\{\mathbb{P}_1,\dots,\mathbb{P}_m\}\in\mathcal{P}\left(\{1,\dots,n\},m\right)}$ in equation (A9) thus runs over all distinct partitions of $\{1,2,\dots,n\}$ into *m* subsets $\mathbb{P}_1,\dots,\mathbb{P}_m$. There is no order to the subsets, so that the partition $\{\{1\},\{2\}\}$ of $\{1,2\}$ is identical to $\{\{2\},\{1\}\}$ and thus considered the same in $\mathcal{P}\left(\{1,2\},2\right)$. We use $\mathbf{K} \left(\mathbb{P}\right)$ to denote sums over momenta given by the indices in set $\mathbb{P}$, equation (81), $\mathbf{K} \left(\mathbb{P}\right) = \sum_{p\in\mathbb{P}} \mathbf{k}_p$. Equation (A9) is a function of the *set* of momenta $\{\mathbf{k}_1,\dots,\mathbf{k}_n\}$, or simply the indices $\{1,\dots,n\}$ alone, and invariant under their permutation.

Equation (A9) will be our induction hypothesis, with the induction to be taken in *n*, the number of outgoing legs. The base cases *n* = 1, *n* = 2 and *n* = 3 are immediately verified, as $\mathcal{P}\left(\{1\},1\right) = \{\,\{\{1\}\}\,\}$ reduces equation (A9) trivially to equation (36), $\mathcal{P}\left(\{1,2\},1\right) = \{\,\{\{1,2\}\}\,\}$ and $\mathcal{P}\left(\{1,2\},2\right) = \{\,\{\{1\},\{2\}\}\,\}$ to equation (A7) and $\mathcal{P}\left(\{1,2,3\},1\right) = \{\,\{\{1,2,3\}\}\,\}$, $\mathcal{P}\left(\{1,2,3\},2\right) = \{\,\{\{1\},\{2,3\}\},\{\{2\},\{3,1\}\},\{\{3\},\{1,2\}\}\,\}$ and $\mathcal{P}\left(\{1,2,3\},3\right) = \{\,\{\{\{1\},\{2\},\{3\}\}\}\,\}$ to equation (A8).

We want to show that if equation (A9) holds for all strictly positive $n\le m-1$ then it also holds for *n* = *m*. To this end we consider two distinct subsets of indices $\mathbb{A}$ and $\mathbb{B}$ with cardinality $|\mathbb{A}|\gt0$ and $|\mathbb{B}|\gt0$ respectively, so that $\mathbb{A}\cap\mathbb{B} = \emptyset$, $\mathbb{A}\cup\mathbb{B} = \{1,\dots,n\}$ and thus $|\mathbb{A}|+|\mathbb{B}| = n$. Each of these sets enters as the argument of diagrams

that have $|\mathbb{A}|$ and $|\mathbb{B}|$ external legs respectively, each parameterised by the momenta given by the subsets, $\{\mathbf{k}_q | q\in \mathbb{A}\}$ and $\{\mathbf{k}_q | q\in \mathbb{B}\}$ respectively. These diagrams can be ‘stitched together’ via the Dean vertex, equation (38), so that

\begin{align*} &amp; \qquad\;\; \, \times \left[ \sum_{a=1}^{|\mathbb{A}|} (-1)^{a-1} (a-1)! \sum_{\{\mathbb{P}_1,\ldots,\mathbb{P}_a\}\in \mathcal{P}\left(\mathbb{A},a\right)} \operatorname{e}^{-D(t-t^{^{\prime}}) \sum_{i=1}^a \mathbf{K} \left(\mathbb{P}_i\right)^2}\right] \nonumber\\ &amp; \qquad\;\; \, \times \left[ \sum_{b=1}^{|\mathbb{B}|} (-1)^{b-1} (b-1)! \sum_{\{\unicode{x211A}_1,\ldots,\unicode{x211A}_b\}\in \mathcal{P}\left(\mathbb{B},b\right)} \operatorname{e}^{-D(t-t^{^{\prime}}) \sum_{j=1}^b \mathbf{K} \left(\unicode{x211A}_j\right)^2}\right] \nonumber\\ &amp; \qquad = n_0\theta(t-t_0) \operatorname{e}^{\mathring{\imath}\mathbf{k}_1+\ldots+\mathbf{k}_n\cdot\mathbf{x}_0} \sum_{a=1}^{|\mathbb{A}|} \sum_{b=1}^{|\mathbb{B}|} (-1)^{a+b} (a-1)! (b-1)! (-2 D \mathbf{K} \left(\mathbb{A}\right) \cdot \mathbf{K} \left(\mathbb{B}\right) ) \nonumber\\ &amp; \qquad\;\; \, \times \sum_{\{\mathbb{P}_1,\ldots\}\in \mathcal{P}\left(\mathbb{A},a\right)} \sum_{\{\unicode{x211A}_1,\ldots\}\in \mathcal{P}\left(\mathbb{B},b\right)} \operatorname{e}^{D(\mathbf{k}_1+\cdots+\mathbf{k}_n)^2 t_0} \operatorname{e}^{-Dt \sum_{i=1}^a \mathbf{K} \left(\mathbb{P}_i\right)^2 } \operatorname{e}^{-Dt \sum_{j=1}^b \mathbf{K} \left(\unicode{x211A}_j\right)^2 } \nonumber \\ &amp; \qquad\;\; \, \times \int_{t_0}^t \mathrm{d}t^{^{\prime}}\, \, \operatorname{exp}\left[ Dt^{^{\prime}}\left( \sum_{i=1}^a \mathbf{K} \left(\mathbb{P}_i\right)^2 + \sum_{j=1}^b \mathbf{K} \left(\unicode{x211A}_j\right)^2 - (\mathbf{k}_1 + \dots + \mathbf{k}_n)^2 \right)\right]. \end{align*}

On the left is a diagram with $|\mathbb{A}|+|\mathbb{B}|$ legs and on the right we use equation (A9) for diagrams with fewer legs, because neither $\mathbb{A}$ nor $\mathbb{B}$ can be empty. If we can show that the sum of all diagrams on the left obeys equation (A9), then the induction step is completed.

Since $\cup_{i = 1}^a\mathbb{P}_i = \mathbb{A}$, we have from equation (81) that $\sum_{i = 1}^a \mathbf{K} \left(\mathbb{P}_i\right) = \mathbf{K} \left(\mathbb{A}\right)$ and similarly $\sum_{j = 1}^b \mathbf{K} \left(\unicode{x211A}_j\right) = \mathbf{K} \left(\mathbb{B}\right)$ and further $\mathbf{K} \left(\mathbb{A}\right)+\mathbf{K} \left(\mathbb{B}\right) = \mathbf{K} \left(\{1,\dots,n\}\right) = \mathbf{k}_1+\dots\mathbf{k}_n$, so that the exponent in square brackets appearing within the *t* ^ʹ^ integral in the last line of equation (A10) can be rearranged as follows:

\begin{align*} &amp; \sum_{i=1}^a \mathbf{K} \left(\mathbb{P}_i\right)^2 + \sum_{j=1}^b \mathbf{K} \left(\unicode{x211A}_j\right)^2 - (\mathbf{k}_1 + \dots + \mathbf{k}_n)^2 \nonumber\\ &amp; \quad = -2 \left( \mathbf{K} \left(\mathbb{A}\right) \cdot \mathbf{K} \left(\mathbb{B}\right) + \sum_{i=1}^a\sum_{e=i+1}^a \mathbf{K} \left(\mathbb{P}_i\right)\cdot\mathbf{K} \left(\mathbb{P}_e\right) + \sum_{j=1}^b\sum_{f=j+1}^b \mathbf{K} \left(\unicode{x211A}_j\right)\cdot\mathbf{K} \left(\unicode{x211A}_f\right) \right) \ , \end{align*}

with the nested double summations generating all cross-terms once. In fact, the bracket on the right hand side of equation (A11) is the sum of the vector products of all $ab+a(a-1)/2+b(b-1)/2 = (a+b)(a+b-1)/2$ distinct pairs of vectors generated with equation (81) from the *a* + *b* sets $\{\mathbb{P}_1,\dots,\mathbb{P}_a,\unicode{x211A}_1,\dots,\unicode{x211A}_b\}$. With equation (A11) we arrive at

The main obstacle to simplify equation (A12) further at this point is the fraction of scalar products of $\mathbf{K} \left({\cdot}\right)$’s. Similar to the three-point case, equation (A8), this will simplify only once we consider the sum over all diagrams with non-equivalent permutations of the external momenta. Since the expressions inserted for sub-diagrams already take care of permutations within each subdiagram we need to consider only different partitionings of the indices $1,\ldots,n$ into subsets $\mathbb{A}$ and $\mathbb{B}$. Diagrammatically, for $n\geqslant2$,

where $\mathbb{A}$ and $\mathbb{B}$ are again the sets of indices of the momenta associated with each part of the partition. The external fields of the sub-diagram labelled $|\mathbb{A}|$ have momenta **k**_*a*_ with $a\in\mathbb{A}$ and correspondingly for the other sub-diagram. The cardinality $|\mathbb{A}|$ of the non-empty partition $\mathbb{A}$, ranges from 1 to *n* − 1. The cardinality of the non-empty partition $\mathbb{B}$ is then given by $|\mathbb{B}| = n - |\mathbb{A}|$. Using equation (A12) for the diagrams summed over in equation (A13), we re-organise the partitioning, as explained below, and rewrite it as

where $\mathcal{F}$ is given by

\begin{align*} &amp; \mathcal{F}(\{\mathbb{P}_1,\dots,\mathbb{P}_a\},\{\unicode{x211A}_1,\dots,\unicode{x211A}_b\}) \nonumber\\ &amp; \quad = \frac{n_0 \theta(t-t_0) \operatorname{e}^{\mathring{\imath}\mathbf{k}_1+\ldots+\mathbf{k}_n\cdot\mathbf{x}_0} (-1)^{a+b}\,(a-1)!\,(b-1)!\,\mathbf{K} \left(\bigcup\limits_{i=1}^a\mathbb{P}_i\right) \cdot \mathbf{K} \left(\bigcup\limits_{i=1}^b\unicode{x211A}_i\right)}{\mathbf{K} \left(\bigcup\limits_{i=1}^a\mathbb{P}_i\right) \cdot \mathbf{K} \left(\bigcup\limits_{i=1}^b\unicode{x211A}_i\right)+ \sum\limits_{i=1}^a\sum\limits_{e=i+1}^a \mathbf{K} \left(\mathbb{P}_i\right)\cdot\mathbf{K} \left(\mathbb{P}_e\right) + \sum\limits_{j=1}^b\sum\limits_{f=j+1}^b \mathbf{K} \left(\unicode{x211A}_j\right)\cdot\mathbf{K} \left(\unicode{x211A}_f\right)} \nonumber\\ &amp; \qquad \times \left[ \mathrm{e}^{-D (t-t_0) (\mathbf{k}_1+\cdots+\mathbf{k}_n)^2} - \mathrm{e}^{-D (t-t_0) \left( \sum_{i=1}^a \mathbf{K} \left(\mathbb{P}_i\right)^2 + \sum_{j=1}^b \mathbf{K} \left(\unicode{x211A}_j\right)^2 \right) } \right] \,. \end{align*}

The parameters *a* and *b* are the cardinalities of the first and the second partition in the argument of $\mathcal{F}$. This function depends on two partitions of two sets, $\mathbb{A}$ and $\mathbb{B}$, and it is invariant under exchange of its two arguments, which are sets of sets. On the basis of the partitions and the globally known $\mathbf{k}_1,\dots,\mathbf{k}_n$, all the vectors on the right of equation (A15) can be constructed, so that $\mathcal{F}$ is solely a function of the two partitions.

Both sides of equation (A14) are performing the same summation, based on the five sums from equations (A12) and (A13). Both summations generate all possible ways of partitioning the *n* external legs into two or more subsets. In fact there is a one-to-one correspondence between every term in the two sums, as we will demonstrate below. The first sum in equation (A14)*a* considers all partitions $\mathcal{P}\left(\{1,\dots,n\},2\right)$ of the full set of indices $\{1,\dots,n\}$ into two sets, $\mathbb{A}$ and $\mathbb{B}$. These indicate the momenta the two subdiagrams shown in equations (A12) and (A13) depend on. To calculate these two subdiagrams all partitions of $\mathbb{A}$ and $\mathbb{B}$ need to be summed over, which is done in the remaining four sums. The right-hand side equation (A14)*b* of equation (A14) performs the same summation, but first produces all partitions of all $\{1,\dots,n\}$ into $m = 2,\dots,n$ non-empty subsets $\{\mathbb{W}_1,\dots,\mathbb{W}_m\}$. In the rightmost sum, these subsets are distributed among the upper and the lower subdiagram by performing a partition into two subsets $\mathbb{T}_A$ and $\mathbb{T}_B$ on the indexing $\{1,\dots,m\}$ of the subsets $\mathbb{W}_t$. These selections of subsets enter into the function $\mathcal{F}$, with, for example, the upper diagram being parameterised by the collection of sets

\begin{align*} \bigcup_{t\in\mathbb{T}_A} \{\mathbb{W}_t\} = \big\{ \mathbb{W}_{t_1}, \mathbb{W}_{t_2},\ldots \big\} \ne \bigcup_{t\in\mathbb{T}_A} \mathbb{W}_t \quad\textrm{for }\quad \mathbb{T}_A=\{t_1,t_2,\ldots\} \ . \end{align*}

Both summations of equation (A14) generate all partitions of the indices and their division into an upper and a lower subdiagram. Any term appearing on the left can be found on the right and vice versa. A set of parameters $\{\mathbb{P}_1,\dots,\mathbb{P}_a\}$ and $\{\unicode{x211A}_1,\dots,\unicode{x211A}_b\}$ on the left is found on the right when $m = a+b$ and $\{\mathbb{W}_1,\dots,\mathbb{W}_m\} = \{\mathbb{P}_1,\dots,\mathbb{P}_a\} \cup \{\unicode{x211A}_1,\dots,\unicode{x211A}_b\}$ are the same partition of $\{1,\dots,n\}$, with exactly one of the partitions $\mathbb{T}_A,\mathbb{T}_B$ of the elements of $\{\mathbb{W}_1,\dots\}$ such that $\cup_{t\in\mathbb{T}_A} \{\mathbb{W}_t\} = \{\mathbb{P}_1,\dots,\mathbb{P}_a\}$ and $\cup_{t\in\mathbb{T}_B} \{\mathbb{W}_t\} = \{\unicode{x211A}_1,\dots,\unicode{x211A}_b\}$ or equivalently $\cup_{t\in\mathbb{T}_A} \{\mathbb{W}_t\} = \{\unicode{x211A}_1,\dots,\unicode{x211A}_b\}$ and $\cup_{t\in\mathbb{T}_B} \{\mathbb{W}_t\} = \{\mathbb{P}_1,\dots,\mathbb{P}_a\}$. Similarly, the term generated by the partition $\cup_{t\in\mathbb{T}_A} \{\mathbb{W}_t\}, \cup_{t\in\mathbb{T}_B} \{\mathbb{W}_t\}$ on the right, can be identified on the left, as the one where $\mathbb{A} = \cup_{t\in\mathbb{T}_A} \mathbb{W}_t$ and $\mathbb{B} = \cup_{t\in\mathbb{T}_B} \mathbb{W}_t$ or vice versa, which are both sets, not partitions. They need to be partitioned subsequently, for example $\mathbb{A}$ into $\{\mathbb{P}_1,\dots,\mathbb{P}_a\} = \cup_{t\in\mathbb{T}_A}\mathbb{W}_t$ and $\mathbb{B}$ into $\{\mathbb{P}_1,\dots,\mathbb{P}_b\} = \cup_{t\in\mathbb{T}_B}\mathbb{W}_t$ or equally $\mathbb{A}$ into $\{\mathbb{P}_1,\dots,\mathbb{P}_a\} = \cup_{t\in\mathbb{T}_B}\mathbb{W}_t$ and $\mathbb{B}$ into $\{\mathbb{P}_1,\dots,\mathbb{P}_b\} = \cup_{t\in\mathbb{T}_A}\mathbb{W}_t$.

Writing $\mathbb{T}_A = \{\alpha_1, \alpha_2, \dots, \alpha_a\}$ and $\mathbb{T}_B = \{\beta_1, \beta_2, \dots, \beta_b\}$, the parameterisation of the right-hand side of equation (A14) allows us to express the denominator of $\mathcal{F}$, equation (A15), succinctly in terms of the new partition $\{\mathbb{W}_1,\dots,\mathbb{W}_m\}$,

\begin{align*} &amp; \mathbf{K} \left( \cup_{t\in\mathbb{T}_A} \{\mathbb{W}_t\}\right) \cdot \mathbf{K} \left( \cup_{t\in\mathbb{T}_B} \{\mathbb{W}_t\}\right) + \sum_{i=1}^a \sum_{e=i+1}^a \mathbf{K} \left(\mathbb{W}_{\alpha_i}\right)\cdot\mathbf{K} \left(\mathbb{W}_{\alpha_e}\right)\nonumber\\ &amp; \quad + \sum_{j=1}^b \sum_{f=j+1}^b \mathbf{K} \left(\mathbb{W}_{\beta_j}\right) \cdot\mathbf{K} \left(\mathbb{W}_{\beta_f}\right) = \sum_{u=1}^m \sum_{v=u+1}^m \mathbf{K} \left(\mathbb{W}_u\right)\cdot\mathbf{K} \left(\mathbb{W}_v\right) \end{align*}

as this is the sum of all cross-terms in the square of $\sum_{t = 1}^m \mathbf{K} \left(\mathbb{W}_t\right)$, as alluded to after equation (A11). Further, the sum of the squares in the exponent of the right-most exponential in equation (A15) can be written as

\begin{align*} \sum_{i=1}^a \mathbf{K} \left(\mathbb{W}_{\alpha_i}\right)^2 + \sum_{j=1}^b \mathbf{K} \left(\mathbb{W}_{\beta_j}\right)^2 = \sum_{u=1}^m \mathbf{K} \left(\mathbb{W}_u\right)^2 \end{align*}

as $\mathbb{T}_A\cup\mathbb{T}_B = \{1,\dots,m\}$. Because the right-hand sides of equations (A17) and (A18) are independent of the partitioning of $\{\mathbb{W}_1,\dots,\mathbb{W}_m\}$ via $\mathbb{T}_A$ and $\mathbb{T}_B$, they can be taken outside the sum over these partitions together with $(-1)^{|\mathbb{T}_A|+|\mathbb{T}_B|} = (-1)^m$,

where we use the shorthands

\begin{align*} \bar{\mathbf{K}}_A &amp;= \mathbf{K} \left( \bigcup\limits_{t\in\mathbb{T}_A} \{\mathbb{W}_t\}\right) = \sum_{t\in\mathbb{T}_A} \mathbf{K} \left(\mathbb{W}_t\right) \end{align*}

\begin{align*} \bar{\mathbf{K}}_B &amp;= \mathbf{K} \left( \bigcup\limits_{t\in\mathbb{T}_B} \{\mathbb{W}_t\}\right) = \sum_{t\in\mathbb{T}_B} \mathbf{K} \left(\mathbb{W}_t\right) ~. \end{align*}

Next we want to simplify the sum in square brackets in equation (A19). Since it is over all ways to partition $\{1,\dots,m\}$ into the two distinct sets that define $\bar{\mathbf{K}}_A$ and $\bar{\mathbf{K}}_B$, we know that the sum over the products $\bar{\mathbf{K}}_A\cdots\bar{\mathbf{K}}_B$ will involve every cross-term $\mathbf{K} \left(\mathbb{W}_u\right)\cdot\mathbf{K} \left(\mathbb{W}_v\right)$ with $u\ne v$ at least once and by symmetry equally often. The sum will therefore cancel with the denominator up to a pre-factor. In order to determine it, we pick a particular scalar product, $\mathbf{K} \left(\mathbb{W}_u\right)\cdot\mathbf{K} \left(\mathbb{W}_v\right)$ for some fixed $u\ne v$ and consider those terms in the sum that contain $\mathbf{K} \left(\mathbb{W}_u\right)\cdot\mathbf{K} \left(\mathbb{W}_v\right)$:

\begin{align*} C_{u,v}&amp; = \mathbf{K} \left(\mathbb{W}_u\right)\cdot\mathbf{K} \left(\mathbb{W}_v\right)\times\sum_{\{\mathbb{T}_A,\mathbb{T}_B\} \in \mathcal{P}\left(\{1,\dots,m\},2\right)} \mathcal{I}\Big((u\in\mathbb{T}_A \land v\in\mathbb{T}_B) \lor (u\in\mathbb{T}_B \land v\in\mathbb{T}_A) \Big) \nonumber\\ &amp; \quad \times (|\mathbb{T}_A|-1)!\,(|\mathbb{T}_B|-1)! \ , \end{align*}

where $\mathcal{I}(\ldots)$ is an indicator function that is 1 only if indices *u* and *v* are in different subsets and 0 otherwise, so that the square bracket in equation (A19) becomes $\sum_{u = 1}^m \sum_{v = u+1}^m C_{u,v}$. We may therefore simply sum over all partitions where *u* and *v* indeed *are* in different subsets, for example *u* in $\mathbb{T}_A$ and *v* in $\mathbb{T}_B$—there is no need to separately consider the case $u\in\mathbb{T}_B$ and $v\in\mathbb{T}_A$, as the resulting partitions are identical. The make-up of the subsets enters only in as far as their cardinalities are concerned, which feature in the factorial. If $n_A = |\mathbb{T}_A|\gt0$ is the cardinality of $\mathbb{T}_A$, that leaves $n_B = m-n_A\gt0$ elements for $\mathbb{T}_B$. With one ‘seat’ in $\mathbb{T}_A$ given to *u*, there are $n_A-1$ further elements to be chosen from the *m* − 2 elements in $\{1,\dots,m\}\setminus\{u,v\}$,

\begin{align*} C_{u,v} &amp;=\mathbf{K} \left(\mathbb{W}_u\right)\cdot\mathbf{K} \left(\mathbb{W}_v\right) \sum_{n_A=1}^{m-1} {m-2 \choose n_A-1} \, (n_A-1)!\, (m-n_A-1)! \nonumber\\ &amp;=\mathbf{K} \left(\mathbb{W}_u\right)\cdot\mathbf{K} \left(\mathbb{W}_v\right) \sum_{n_A=1}^{m-1} (m-2)!\, =(m-1)!\,\mathbf{K} \left(\mathbb{W}_u\right)\cdot\mathbf{K} \left(\mathbb{W}_v\right) \ , \end{align*}

which means that the square bracket in equation (A19) cancels with the denominator in the preceding fraction up to a factor $(m-1)!$, which can be taken outside the second sum,

The first exponential in the final bracket is independent of the partition, so that the sum degenerates into the count of the ways a set of *n* elements can be partitioned into *m* non-empty sets, given by the Stirling number of the second kind, $\left\{ \begin{array}{c} n \\ m \end{array} \right\}$. We find with the help of [28, 9.745.1]

\begin{align*} \sum_{m=2}^n (-1)^m\,(m-1)! \sum_{\{\mathbb{W}_1,\dots,\mathbb{W}_m\} \in \mathcal{P}\left(\{1,\dots,n\},m\right)} 1 = \sum_{m=2}^n (-1)^m\,(m-1)!\, \left\{ \begin{array}{c} n \\ m \end{array} \right\} = 1 \ . \end{align*}

With that in place, we rewrite equation (A23) as

The exponential of $-D (t-t_0) (\mathbf{k}_1+\cdots+\mathbf{k}_n)^2$ in the curly brackets is the summand of the subsequent sum running over $m\geqslant2$ evaluated for *m* = 1, because the only partition of $\{1,\dots,n\}$ into *m* = 1 subsets is $\mathbb{W}_1 = \{1,\dots,n\}$ which produces the vector $\mathbf{K} \left(\mathbb{W}_1\right) = \mathbf{k}_1+\cdots+\mathbf{k}_n$. It follows that

which is equation (A9). We have thus demonstrated that if equation (A9) holds for all diagrams with fewer than $n\geqslant2$ legs, as they enter into equation (A10), then equation (A9) also holds for the diagrams with *n* legs. This concludes the induction step and together with the base case *n* = 1 proves equations (A9) and (80) for all $n\geqslant1$.

## Appendix B Multiple starting points

We generalise our calculation of the connected and full moments of the integrated particle number density in Dean’s formalism, section 5, to the case where a total of *n*_0_ particles are initialised at $H \leqslant n_0$ distinct sites. We upgrade the previous derivation by replacing in the action equations (24) and (79) and correspondingly in equations (26) and (80)

\begin{align*} n_0 \operatorname{e}^{i\mathbf{k}_0 \mathbf{x}_0} \qquad \textrm{by} \qquad \sum_{h=1}^{H} n_{0,h} \operatorname{e}^{i \mathbf{k}_0 \mathbf{x}_{0,h}} \end{align*}

where $x_{0,h}$ for $h = 1,\ldots,H$ denote the $n_{0,h}\in \mathbb{N}$ particles’ initial positions such that

\begin{align*} \sum_{h=1}^H n_{0,h} = n_0~. \end{align*}

In section 5 and appendix A we were entirely concerned with connected diagrams, where $n_0\operatorname{e}^{\mathring{\imath} \mathbf{x}_0\cdot\mathbf{k}_0}$ only ever enters linearly. Replacing it according to equation (B1) renders each such diagram a sum over the *H* distinct locations, each such sum still to be considered a single *connected* diagram. This equally applies to the central result equation (88), which now reads

where $I_{\Omega,h}(t-t_0)$ denotes the transition probability from the starting point $x_{0,h}$ into the set Ω.

The full moments of the integrated particle number density for distinct starting points can also be derived straightforwardly following the calculation in equations (90)–(100). Using equation (B3) for the associated connected moments, we arrive at

\begin{align*} &amp; \quad\! = \sum_{j_1+\cdots+j_H=n} \binom{n}{j_1,\ldots,j_H} \prod_{h=1}^H \left. \frac{\mathrm{d}^{j_h}}{\mathrm{d}z^{j_h}} \right|_{z=0} \left[1 + (\mathrm{e}^z-1)I_{\Omega,h}\right]^{n_{0,h}} \end{align*}

\begin{align*} &amp; \quad\! = \sum_{j_1+\cdots+j_H=n} \binom{n}{j_1,\ldots,j_H} \prod_{h=1}^H \sum_{k_h=0}^{n_{0,h}} {n_{0,h} \choose k_h} \left(I_{\Omega,h}\right)^{k_h} k_h! \left\{ \begin{array}{c} j_h \\ k_h \end{array} \right\} \ , \end{align*}

where we have used the generalised product rule to go from equation (B4) to (B5) by swapping the differential operator into the product, as well as the intermediate result equations (97)–(102) for the last step. One can easily verify that equation (B6) reduces to equation (102) when *H* = 1, i.e. when all particles are initialised at the same point.

To show particle entity we use a similar procedure as in the single source case from equation (102) to (106),

where the second sum on the right runs over all non-negative integers $j_1,j_2,\ldots,j_H$ which sum to *n*. We now use that $\sum_{n = 0}^\infty \sum_{j_1+\cdots+j_H = n} = \sum_{j_1 = 0}^\infty \ldots \sum_{j_H = 0}^\infty$ as the sums converge individually and absolutely, and equation (103) to obtain

\begin{align*} &amp; \qquad\quad = \prod_{h=1}^H \left( \sum_{j_h = 0}^\infty \sum_{k_h=0}^{n_{0,h}} \binom{n_{0,h}}{k_h} (1-I_{\Omega,h})^{n_{0,h}-k_h} (I_{\Omega,h} )^{k_h} \frac{(2 \pi \mathring{\imath} k_h)^{j_h}}{j_h !} \right) \end{align*}

\begin{align*} &amp; \qquad\quad = \prod_{h=1}^H \left( \sum_{k_h=0}^{n_{0,h}} \binom{n_{0,h}}{k_h} (1-I_{\Omega,h})^{n_{0,h}-k_h} (I_{\Omega,h} )^{k_h} \mathrm{e}^{2 \pi \mathring{\imath} k_h} \right) \end{align*}

\begin{align*} &amp; \qquad\quad = \prod_{h=1}^H \left( 1\right) \end{align*}

\begin{align*} &amp; \qquad\quad = 1 \, . \end{align*}

This completes the derivation of particle entity according to the criterion equations (64) for particles initialised as multiple origins, cf equation (106).

## Appendix C Integer requirements on initialisation amplitude *n*_0_

The *n*th full moment, in the form of the right hand side equation (102), has an instructive physical interpretation drawing on *n*_0_ being integer. To see this, we write the *n*th moment of the particle number as

\begin{align*} {\left\langle \left( \int_\Omega \mathrm{d}^{d}x \rho(\mathbf{x},t)\right)^n \right\rangle} = \int_\Omega \mathrm{d}^{d}{x^{^{\prime}}_1}\mathrm{d}^{d}{x^{^{\prime}}_2}\ldots\mathrm{d}^{d}{x^{^{\prime}}_n} {\left\langle \rho(\mathbf{x}^{^{\prime}}_1,t) \rho(\mathbf{x}^{^{\prime}}_2,t)\ldots \rho(\mathbf{x}^{^{\prime}}_n,t) \right\rangle} \end{align*}

with each density $\rho(\mathbf{x}^{^{\prime}}_i,t)$ considered a random variable as a function of the positions $x_j(t)$ of *n*_0_ particles indexed by $j = 1,2,\ldots,n_0$

\begin{align*} \rho(\mathbf{x}^{^{\prime}}_i,t) = \sum_{j=1}^{n_0} \delta(\mathbf{x}_i^{^{\prime}}-\mathbf{x}_j(t)) \ , \end{align*}

generating $n_0^n$ terms of products of *δ*-functions in equation (C1). To calculate the right-hand side of equation (C1) on the basis of equation (C2) using equation (87) in the form

\begin{align*} \int_\Omega \mathrm{d}^{d}x^{^{\prime}} {\left\langle \delta(\mathbf{x}^{^{\prime}}-\mathbf{x}_j(t) \right\rangle} = I_\Omega(t-t_0) \end{align*}

requires careful bookkeeping of how often each particle coordinate $\mathbf{x}_j(t)$ is repeated. For example

\begin{align*} \int_\Omega \mathrm{d}^{d}{x^{^{\prime}}_1}\mathrm{d}^{d}{x^{^{\prime}}_2} {\left\langle \delta(\mathbf{x}^{^{\prime}}_1-\mathbf{x}_1(t))\delta(\mathbf{x}^{^{\prime}}_2-\mathbf{x}_2(t)) \right\rangle} &amp;= I_\Omega^2 \end{align*}

\begin{align*} \int_\Omega \mathrm{d}^{d}{x^{^{\prime}}_1}\mathrm{d}^{d}{x^{^{\prime}}_2} {\left\langle \delta(\mathbf{x}^{^{\prime}}_1-\mathbf{x}_1(t))\delta(\mathbf{x}^{^{\prime}}_2-\mathbf{x}_1(t)) \right\rangle} &amp;= I_\Omega \end{align*}

as particle coordinates are independent in equation (C4*a*), but not in equation (C4*b*), where in fact $\delta(\mathbf{x}^{^{\prime}}_1-\mathbf{x}_1(t))\delta(\mathbf{x}^{^{\prime}}_2-\mathbf{x}_1(t)) = \delta(\mathbf{x}^{^{\prime}}_1-\mathbf{x}_1(t))\delta(\mathbf{x}^{^{\prime}}_2-\mathbf{x}^{^{\prime}}_1)$. To calculate the right-hand side of equation (C1) thus is a matter of allowing for $k = 1,2,\ldots,n$ distinct particle coordinates $x_j(t)$ from each of the *n* sums equation (C2). There are $n_0(n_0-1)\cdot\ldots\cdot(n_0-k+1) = {n_0\choose k}k!$ such choices. As each of the $\rho(\mathbf{x}_i^{^{\prime}},t)$ is a function of a different dummy variable, they have to be distributed among the *k* distinct particle coordinates. There are $\left\{ \begin{array}{c} n \\ k \end{array} \right\}$ choices for that. The integration produces $I_\Omega^k$ depending on the number *k* of distinct particle coordinates following the reasoning for equation (C4). In summary, we arrive at

\begin{align*} {\left\langle \left( \int_\Omega \mathrm{d}^{d}x \rho(\mathbf{x},t)\right)^n \right\rangle} = \sum_{k=0}^n {n_0\choose k} I_\Omega^k k! \left\{ \begin{array}{c} n \\ k \end{array} \right\} \ , \end{align*}

including *k* = 0 in the summation to cover the special case of *n* = 0. Equation (C5) is subtly different from equation (102), as the upper limit of the sum in equation (102) is *n*_0_, while it is *n* in equation (C5). However, $\left\{ \begin{array}{c} n \\ k \end{array} \right\}$ in equation (102) vanishes if *k* exceeds *n* and ${n_0\choose k}$ in equation (102) vanishes if *integer* *k* exceeds *integer* *n*_0_, i.e. in both sums the upper limit may be replaced by $\min{(n,n_0)}$, provided only *n*_0_ is integer. We conclude that the full moment equation (102) has a sensible interpretation in terms of particle numbers only if *n*_0_ is integer.

The key ingredient here are the falling factorials ${n_0\choose k}k! = n_0(n_0-1)\cdot\ldots\cdot(n_0-k+1)$. We have seen how they emerge in Dean’s theory by computing the connected moments, equation (102) and also equation (C5), explicitly. This structure is another signature of particle entity, in the sense that it highlights the special role played by the integer nature of the initial particle number *n*_0_. Unsurprisingly, it similarly emerges in Doi–Peliti. In particular, the full moments of the particle number at position **x** are given by

where $\hat{A}$ denotes the time evolution operator of equation (4). We then use

\begin{align*} \big(a^\dagger(\mathbf{x}_0)\big)^{n_0} = \big(\tilde{a}(\mathbf{x}_0) + 1\big)^{n_0} = \sum_{k=0}^{n_0} {n_0 \choose k} \big(\tilde{a}(\mathbf{x}_0)\big)^k \end{align*}

together with [29]

\begin{align*} \left\langle \unicode{x263C} \right| (a^\dagger(\mathbf{x}) a(\mathbf{x}))^n = \sum_{\ell=0}^n \left\{ \begin{array}{c} n \\ \ell \end{array} \right\} \left\langle \unicode{x263C} \right| \big(a(\mathbf{x}) \big)^\ell \end{align*}

to rewrite the right-hand side of equation (C6) as

\begin{align*} &amp; \left\langle \unicode{x263C} \right| (a^\dagger(\mathbf{x}) a(\mathbf{x}))^n \operatorname{e}^{\hat{A}(t-t_0)} (a^\dagger(\mathbf{x}_0))^{n_0} \left| 0 \right\rangle\nonumber\\ &amp; \quad = \sum_{\ell=0}^n \left\{ \begin{array}{c} n \\ \ell \end{array} \right\} \sum_{k=0}^{n_0} {n_0 \choose k} \left\langle \unicode{x263C} \right| \big(a(\mathbf{x})\big)^\ell \operatorname{e}^{\hat{A}(t-t_0)} \big(\tilde{a}(\mathbf{x}_0)\big)^k \left| 0 \right\rangle \end{align*}

\begin{align*} &amp; \quad = \sum_{\ell=0}^{n_0} \left\{ \begin{array}{c} n \\ \ell \end{array} \right\} {n_0 \choose \ell} \ell! \left\langle \unicode{x263C} \right| a(\mathbf{x}) \operatorname{e}^{\hat{A}(t-t_0)} \tilde{a}(\mathbf{x}_0) \left| 0 \right\rangle^\ell \end{align*}

where we have used $\left\langle \unicode{x263C} \right| (a(\mathbf{x}))^\ell \operatorname{exp}(\hat{A}(t-t_0))(\tilde{a}(\mathbf{x}_0))^k \left| 0 \right\rangle = \delta_{k\ell} \ell! \left\langle \unicode{x263C} \right| a(\mathbf{x}) \operatorname{exp}(\hat{A}(t-t_0)) \tilde{a}(\mathbf{x}_0) \left| 0 \right\rangle^\ell$ in the absence of interactions. The final expression corresponds to the one we found via the Dean route, equation (102), with $I_\Omega(t-t_0)$ replaced by the Doi–Peliti propagator $\left\langle \unicode{x263C} \right| a(\mathbf{x}) \operatorname{exp}\hat{A}(t-t_0)) \tilde{a}(\mathbf{x}_0) \left| 0 \right\rangle$.
